# Reconsidering gas as clean energy: Switching to electricity for household cooking to reduce NO_2_-attributed disease burden

**DOI:** 10.1016/j.eehl.2023.10.003

**Published:** 2023-11-17

**Authors:** Ying Hu, Ye Wang, Zhuohui Zhao, Bin Zhao

**Affiliations:** a Department of Building Science, School of Architecture, Tsinghua University, Beijing 100084, China; b School of Public Health, Fudan University, Shanghai 200433, China; c Key Laboratory of Public Health Safety of the Ministry of Education, NHC Key Laboratory of Health Technology Assessment, Fudan University, Shanghai 200433, China; d Shanghai Typhoon Institute/CMA, Shanghai Key Laboratory of Meteorology and Health, IRDR International Center of Excellence on Risk Interconnectivity and Governance on Weather/Climate Extremes Impact and Public Health, WMO/IGAC MAP-AQ Asian Office Shanghai, Fudan University, Shanghai 200433, China; e Beijing Key Laboratory of Indoor Air Quality Evaluation and Control, Tsinghua University, Beijing 100084, China

**Keywords:** Environmental risk, Indoor air pollution, Nitrogen dioxide, Health effect, Cooking

## Abstract

Nitrogen dioxide (NO_2_) is a prevalent air pollutant in urban areas, originating from outdoor sources, household gas consumption, and secondhand smoke. The limited evaluation of the disease burden attributable to NO_2_, encompassing different health effects and contributions from various sources, impedes our understanding from a public health perspective. Based on modeled NO_2_ exposure concentrations, their exposure–response relationships with lung cancer, chronic obstructive pulmonary disease, and diabetes mellitus, and baseline disability-adjusted life years (DALYs), we estimated that 1,675 (655–2,624) thousand DALYs were attributable to NO_2_ in urban China in 2019 [138 (54–216) billion Chinese yuan (CNY) economic losses]. The transition from gas to electricity for household cooking was estimated to reduce the attributable economic losses by 35%. This reduction falls within the range of reductions achieved when outdoor air meets the World Health Organization interim target 3 and air quality guidelines for annual NO_2_, highlighting the significance of raising awareness of gas as a polluting household energy for cooking. These findings align with global sustainable development initiatives, providing a sustainable solution to promote public health while potentially mitigating climate change.

## Highlights

• NO_2_-attributed DALYs for lung cancer, COPD, and diabetes mellitus were estimated.
• Human exposure to NO_2_ from gas cooking, second-hand smoke, and outdoor sources was considered.
• 1,675 (655–2,624) thousand DALYs were attributable to NO_2_ exposure in urban China in 2019.
• Gas-to-electricity switching for household cooking reduce the attributable economic losses by 35%.

## Graphical abstract

**Figure:**
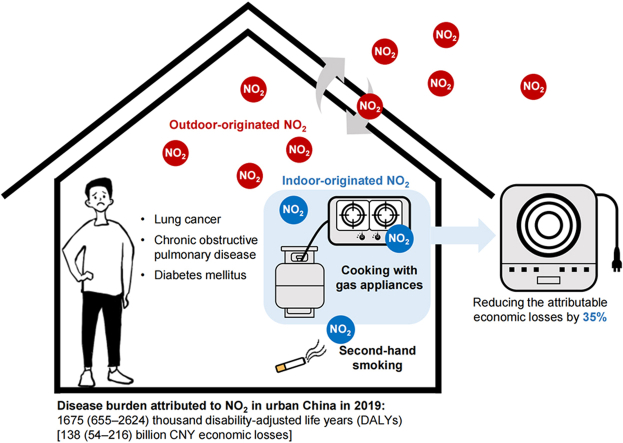

## 1 Introduction

Air pollution is a major global concern for public health [1]. Although countries worldwide have been fighting outdoor air pollution for years [2], indoor air pollution has recently been under the spotlight because of its comparable disease burden to that of outdoor pollution [3]. Besides the migration of outdoor air pollutants indoors, household fossil fuel consumption is a major source of indoor air pollution. While residential energy consumption in rural areas is undergoing a transition from solid fuel to gas fuel and electricity [4], the consumption of gas fuel for cooking remains common in urban households [5]. Various types of gas fuels, such as natural gas, liquefied petroleum gas (LPG), coal gas, and other alternative options, are employed, all emitting air pollutants into the indoor environment [6]. With the concurrent trends of population growth and urbanization [7], the recognition of public health challenges arising from air pollution in urban areas, coupled with the corresponding policy initiatives, is steadily gaining prominence. Among air pollutants originating from both outdoor air and household gas consumption in urban areas, nitrogen dioxide (NO_2_) is prominent because of its significant emissions and health effects.

NO_2_ in the atmosphere primarily originates from combustion sources, such as the transport and power industries [8]. Urban areas, typically characterized by heavy traffic, experience severe ambient NO_2_ pollution. In 2019, the global annual average surface concentration of NO_2_ in urban areas was 22 μg/m^3^ [9], exceeding the air quality guideline (AQG) of 10 μg/m^3^ recommended by the World Health Organization (WHO) [2]. Because urban residents spend most of their time indoors [10,11], indoor combustion processes, including household gas consumption and secondhand smoke, account for 30%–40% of the NO_2_ exposure by urban residents and lead to high concentrations of NO_2_ indoors [12]. Breathing in high concentrations of NO_2_ lead to oxidative injuries in the airways, which may result in asthma [13], chronic obstructive pulmonary disease (COPD) [14], lung cancer (LC) [15], and even diabetes mellitus (DM) [16]. In China, a 10-μg/m^3^ increase in the 2-day moving average of NO_2_ concentrations is significantly associated with a 0.9% increase in mortality from total nonaccidental causes, which is higher than the estimated 0.22% increase associated with fine particulate matter (PM_2.5_) [17,18]. Recent studies have assessed the disease burden attributable to outdoor NO_2_, including premature mortality [[19], [20], [21], [22]], non-communicable disease morbidity [23], and pediatric asthma [9,[24], [25], [26], [27]]. However, the indoor sources of NO_2_ have been largely overlooked, except for one study that found a significant contribution from both indoor and outdoor sources of NO_2_ to the burden of pediatric asthma [27].

A comprehensive evaluation of the disease burden attributable to NO_2_, encompassing its health effects and contributions from both indoor and outdoor sources, is crucial for understanding the current state of NO_2_ pollution from a public health perspective. Moreover, targeted control strategies for NO_2_ from various sources, including outdoor sources, household gas consumption, and secondhand smoke, may be effective in mitigating NO_2_ pollution [28]. However, the effectiveness of source-specific control measures in reducing the burden of diseases attributable to NO_2_ has yet to be quantified.

Given that urban areas in China currently house approximately 10% of the world's population and suffer from high levels of both outdoor and indoor NO_2_ pollution, exploring the public health issues related to NO_2_ in these areas is of paramount importance. In this study, we estimated the burden of diseases attributable to NO_2_ from indoor and outdoor sources in urban areas in China in 2019, and the burden reduction by restrictions on NO_2_ emissions indoors and outdoors. The disease burden of LC, COPD, and DM was reported in disability-adjusted life years (DALYs: a combination of both the years of potential life lost due to premature mortality and years of productive life lost due to a disability) and the loss of economic production value due to DALYs.

## 2 Methods

### 2.1 Overview

**Fig. 1:**
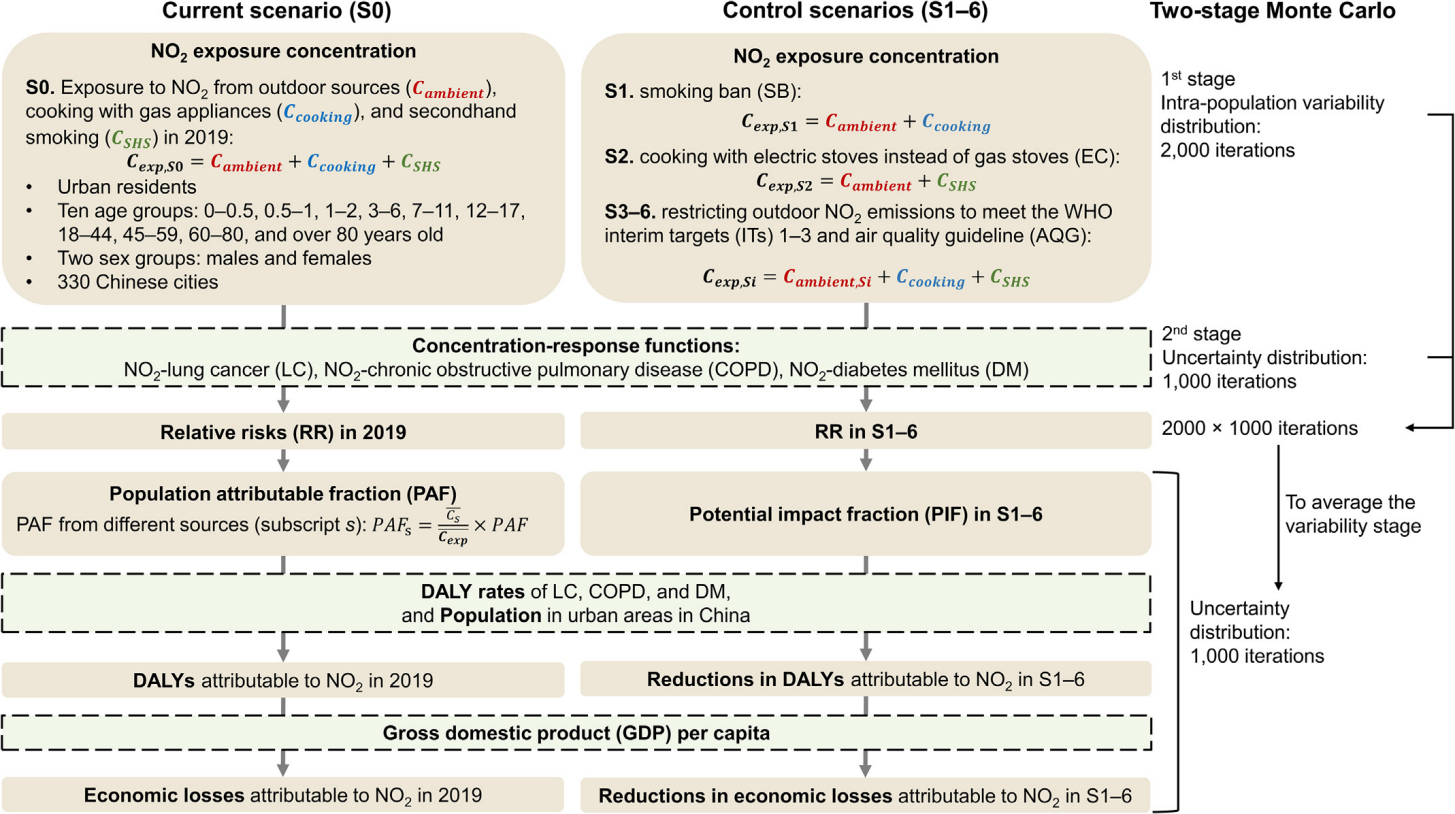
Framework of methods.

The methodological framework is illustrated in Fig. 1. First, we estimated the NO_2_ exposure concentrations in the current and control scenarios. Then, we calculated the DALYs attributable to NO_2_ in the current scenario and the reduction in DALYs in the control scenarios based on NO_2_ exposure concentration, concentration–response functions, baseline DALY rates, and population data in urban China. The DALYs attributable to NO_2_ were monetized according to the gross domestic product (GDP) per capita in urban China. To capture uncertainty intervals (UI), a two-stage Monte Carlo method was employed to estimate NO_2_-attributed DALYs and economic losses.

The DALYs and economic losses attributable to NO_2_ are influenced by multiple factors, some of which exhibit regional and populational variations. Thus, we estimated the disease burden attributable to NO_2_ in urban areas in 330 Chinese cities, among 10 age groups, for both males and females, aiming to identify and explain the differences. Among 330 cities, particular attention was given to first-tier and new first-tier cities in China—highly developed cities based on multiple criteria such as population, economic development, cultural significance, and future prospects.

### 2.2 Estimation of exposure

NO_2_ is an air pollutant with notable sources in both indoor and outdoor environments [29]. Outdoor NO_2_ sources lead to exposure during both indoor and outdoor activities due to the infiltration of outdoor NO_2_ into indoor spaces. Conversely, indoor NO_2_ sources are associated with NO_2_ emissions from gas and tobacco combustion, resulting in exposure during indoor activities. The NO_2_ exposure concentration represents the average concentration of NO_2_ in the air inhaled by an individual. It is determined by computing a weighted average of NO_2_ concentrations in different micro-environments, considering the time spent in these settings and the respiratory rates during various activities. To quantify NO_2_ exposure concentrations from a specific source, the NO_2_ concentrations within various micro-environments originating from that source should be considered.

To estimate the NO_2_ exposure concentrations from different sources, we have developed a validated source-specific model based on the kinetic law of NO_2_ migration, emission, and deposition, as well as human activities. The model and its validation are detailed in our previous study by Hu and Zhao [12], with the essential information described in the Supplemental experimental procedures. The model considers various input parameters, including outdoor NO_2_ concentrations from monitoring stations, the emission rates of NO_2_ from gas cooking and smoking, and habits related to cooking, smoking, ventilation, and outdoor activities. Using this model, we obtained indoor and outdoor NO_2_ exposure concentrations from various sources (i.e., outdoor sources, gas cooking, and secondhand smoking) for urban residents of different ages (ten age groups: 0–0.5, 0.5–1, 1–2, 3–6, 7–11, 12–17, 18–44, 45–59, 60–80, and over 80 years old), sexes (male and female), and cities (330 Chinese cities) in China under multiple scenarios.

We set up seven scenarios to gain insights into the current NO_2_ exposure concentrations among urban residents in China and evaluate the effectiveness of source control measures in reducing NO_2_ exposure. The NO_2_ exposure concentrations from outdoor sources, gas cooking, and secondhand smoking in these scenarios were denoted as *C*_*ambient*_, *C*_*cooking*_, and *C*_*SHS*_, respectively. These scenarios included (shown in Table S2):

S0: Current scenario in 2019. *C*_*ambient*_, *C*_*cooking*_, and *C*_*SHS*_ were obtained from our previous study as mentioned before [12];

S1: Smoking ban (SB), smoking is prohibited indoors (*C*_*SHS*_ = 0; *C*_*cooking*_ and *C*_*ambient*_ were equal to those in 2019);

S2: Cooking with electric stoves instead of gas stoves in residences (EC), all residents use electric stoves for cooking in Chinese urban areas, and electrical cooking appliances hardly produced NO_2_ (*C*_*cooking*_ = 0; *C*_*SHS*_ and *C*_*ambient*_ were equal to those in 2019).

S3–6: Restricting outdoor NO_2_ emissions to meet the WHO interim targets (ITs) 1–3 and AQG for annual NO_2_ concentrations issued in 2021. *C*_*SHS*_ and *C*_*cooking*_ in S3–6 were equal to those in 2019, and *C*_*ambient*_ in S3–6 was calculated as follows:

(1) $$\begin{array}{c} C_{ambient}=\left\{ \begin{array}{c} C_{ambient}\;in\;2019\;\;\;\;\;\;\;\;\;for\;Outdoor\;concentration\;in\;2019\;\leq \;Target\\ Target\;\times \;f_{\exp}\;\;\;\;\;\;\;\;\;for\;Outdoor\;concentration\;in\;2019\;>\;Target \end{array}\right. \end{array}$$

where *Target* is the target annual NO_2_ concentration and is 40 μg/m^3^ (IT1), 30 μg/m^3^ (IT2), 20 μg/m^3^ (IT3), and 10 μg/m^3^ (AQG) for S3–6, respectively. *f*_*exp*_ is the exposure factor, which is defined as the ratio of the actual inhaled outdoor-originated NO_2_ concentration to the outdoor NO_2_ concentration [30]. The value of *f*_*exp*_ was less than 1 because of the surface removal of NO_2_ indoors, and was influenced by air exchange between indoors and outdoors, as well as the time people spend indoors and outdoors, resulting in variations across different regions. *f*_*exp*_ in 31 Chinese provinces was estimated and verified in our previous study (shown in Table S7) [30]. The mean and standard deviation of the NO_2_ exposure concentrations in 2019 are provided in Table S13 in our previous study by Hu and Zhao [12] as mentioned before, and the NO_2_ exposure concentrations under the seven scenarios are presented in Figs. S6 and S7.

### 2.3 Concentration–response functions

In this study, we derived concentration–response functions from a meta-analysis conducted by Chen et al. [31]. Compared to other meta-analyses, Chen et al. reviewed the highest number of studies (81 studies mainly performed in China, Europe, and North America). With approximately half of the reviewed studies conducted in China, the review significantly minimized uncertainty when applying the concentration–response function to an urban Chinese population in this study. The review also extended over a broader range of publication years, spanning from 1980 to 2019. Notably, it included studies on the health effects of indoor and ambient NO_2_ exposure, which is most relevant for analyzing the disease burden attributable to overall indoor and outdoor NO_2_ exposure. The meta-analysis revealed that NO_2_–outcome pairs, including NO_2_–pediatric asthma, NO_2_–COPD, NO_2_–DM, NO_2_–LC, and NO_2_–preterm birth, were robust and reliable, with no publication bias. We selected three diseases, i.e., LC, COPD, and DM, to analyze the disease burden attributable to NO_2_, as these diseases have a significant impact on public health and are the leading causes of DALYs. The relative risks (*RRs*), which are the ratio of the probability of developing a disease when exposed to a certain concentration of NO_2_ to the probability of developing the disease in the non-exposed group, were calculated as follows according to the meta-analyses [31]:

(2) $$RR=\left\{ \begin{array}{c} {{RR}_{0}}^{\frac{C_{\exp}}{\Delta C_{0}}}\;\;\;\;\;\;\;\;\;\;\;for\;C_{\exp}\;\leq \;MaxC\\ {{RR}_{0}}^{\frac{MaxC}{\Delta C_{0}}}\;\;\;\;\;\;\;\;for\;C_{\exp}\;>\;MaxC \end{array}\right.$$

(3) $$\begin{array}{c} C_{\exp}=C_{ambient}+C_{cooking}+C_{SHS} \end{array}$$

where *C*_*exp*_ (μg/m^3^) is the annual average NO_2_ exposure concentration, *RR*_0_ is the relative risk per unit increase in NO_2_ exposure concentrations, Δ*C*_0_ (μg/m^3^) is the unit of increase, and *MaxC* (μg/m^3^) is the maximal level of NO_2_ exposure in the meta-analysis. Eq. 2 means the conservation estimation of *RR*s when the exposure concentrations reach or exceed *MaxC*s, since the extrapolation of concentration–response relationships lacked epidemiological evidence and could potentially result in unrealistically high *RR*s [31]. In this study, Δ*C*_0_ was 10 μg/m^3^, and *RR*_0_ and *MaxC* were 1.055 (1.010–1.101) and 54.0 μg/m^3^ for LC, 1.016 (1.012–1.020) and 60.7 μg/m^3^ for COPD, and 1.019 (1.009–1.029) and 44.0 μg/m^3^ for DM [31].

### 2.4 DALY and economic loss estimation

We estimated the DALYs attributable to NO_2_ from three sources (i.e., outdoor sources, gas cooking, and secondhand smoking) due to three diseases (LC, COPD, and DM) in the current scenario and the reduction in DALYs in control scenarios. The estimation was based on the population-attributable fraction, baseline DALY rate of the three diseases, and population in urban areas, using the following equation:

(4) $$\begin{array}{c} {DALY}_{s,d,g}={PAF}_{s,d,g}\times {DALY\;rate}_{g,d}\times N_{g} \end{array}$$

where *PAF* refers to the population-attributable fraction, which is the proportion of incidence in a population that can be attributed to exposure to NO_2_. *DALY rate* is the DALYs per 100,000 people, and *N* is the population. Subscript *s* denotes the source of NO_2_, subscript *d* denotes the type of disease, and subscript *g* denotes the group of people from a specific age and sex group in a specific city. The *DALY rate* and *N* for people from each group *g* are detailed in the Supplemental experimental procedures. *PAF* was calculated according to the following equation [1]:

(5) $$\begin{array}{c} {PAF}_{d,g}=\frac{\bar{{RR}_{S0,d,g}}\;-\;1}{\bar{{RR}_{S0,d,g}}} \end{array}$$

where $\bar{{RR}_{S0}}$ is the average relative risk of the simulated individuals to develop disease when exposed to NO_2_ in the current scenario. To differentiate *PAF* from each source, we divided *PAF* according to the proportion of exposure from each source, using the method employed in the Global Burden of Disease Study 2019 to apportion the disease burden attributable to PM_2.5_ from household air pollution and ambient air pollution [1]:

(6) $$\begin{array}{c} {PAF}_{s,d,g}=\frac{\bar{C_{s,g}}}{\bar{C_{\exp,g}}}\times {PAF}_{d,g} \end{array}$$

where $\bar{C_{s}}$ (μg/m^3^) is the average of NO_2_ exposure concentration from source *s*, $\bar{C_{\exp}}$ (μg/m^3^) is the average of NO_2_ exposure concentration from all sources. The reduction of DALYs in control scenarios S1–6 (*RDALY*_*Si*_) was calculated as follows:

(7) $$\begin{array}{c} {RDALY}_{Si,g,d}={PIF}_{Si,g,d}\times {DALY\;rate}_{g,d}\times N_{g} \end{array}$$

(8) $$\begin{array}{c} {PIF}_{Si,g,d}=\frac{\bar{{RR}_{S0,d,g}}-\bar{{RR}_{Si,g,d}}}{\bar{{RR}_{S0,d,g}}} \end{array}$$

where *PIF*_*Si*_ is the potential impact fraction [32] of the control strategy in scenario *Si*, and $\bar{{RR}_{Si}}$ is the average relative risk in scenario *Si*.

To provide a measure that is easily relatable to policymakers and commonly used in economic assessments of disease burden, we estimated economic losses (*EL*) or reductions of economic losses (*REL*) using the human capital approach under the assumption that one DALY is equal to one GDP per capita (*GDP*_*p*_) loss [33]:

(9) $$\begin{array}{c} EL\;or\;REL=GDP_{p}\times \left(DALY\;or\;RDALY_{Si}\right) \end{array}$$

*GDP*_*p*_ in 330 Chinese cities in 2019 is presented in Table S5.

### 2.5 Uncertainty analysis

We used a two-stage Monte Carlo [34] approach to model the distribution of DALYs and economic losses attributable to NO_2_. The first stage involved 2,000 iterations to capture the intra-population variability in the distribution of NO_2_ exposure concentrations. The second stage involved 1,000 iterations to account for the uncertainty distribution of the concentration–response functions and DALY rates. The total number of iterations in the Monte Carlo simulation was 2,000,000 and was found to be robust, as shown in Supplemental experimental procedures. We calculated the population-level average for the variability stage of the *RR* and exposure concentration (*C*) to obtain $\bar{RR}$ and $\bar{C}$, respectively. We then generated 1,000 DALYs and economic losses for each group of individuals in each scenario and computed the mean and 95% uncertainty distribution (2.5th–97.5th percentile) of the 1,000 iterations.

## 3 Results

### 3.1 The burden of diseases in 2019

In 2019, the population-weighted NO_2_ exposure concentration was 26.7 μg/m^3^ (95% confidence interval: 9.0–57.1 μg/m^3^) in urban areas in China, exceeding the WHO AQG of an annual mean concentration of NO_2_ (10 μg/m^3^). The DALYs attributable to NO_2_ were 1,675 (655–2,624) thousand in urban areas in China, including 64% for LC, 20% for COPD, and 16% for DM (Table 1). The total NO_2_-attributed DALYs were equivalent to 138 (54–216) billion Chinese yuan (CNY) economic losses, equivalent to a thousandth of China's GDP in 2019.

**Table 1:** DALYs and economic losses attributable to NO_2_ in urban areas in China.

Current scenario^a^		DALYs (thousand)				Economic losses (billion CNY)			
	Source	LC	COPD	DM	Total	LC	COPD	DM	Total
S0 (2019)	Total	1,070 (265–1,808)	345 (262–428)	260 (128–389)	1675 (655–2,624)	88 (22–149)	28 (21–35)	22 (11–33)	138 (54–216)
	Gas cooking	415 (103–702)	135 (103–168)	107 (52–159)	657 (258–1,030)	32 (8–54)	10 (8–13)	8 (4–13)	51 (20–79)
	SHS	9 (2–16)	2 (2–3)	2 (1–3)	14 (5–22)	0.8 (0.2–1.3)	0.2 (0.2–0.3)	0.2 (0.1–0.3)	1.2 (0.4–1.9)
	Ambient	645 (160–1,090)	207 (157–256)	151 (74–226)	1,004 (392–1,573)	56 (14–94)	18 (13–22)	13 (7–20)	86 (34–135)
Control scenario^b^		Reductions in DALYs (thousand)				Reductions in economic losses (billion CNY)			
		LC	COPD	DM	Total	LC	COPD	DM	Total
S1 (SB)		10 (2–17)	3 (2–3)	2 (1–3)	15 (5–23)	0.8 (0.2–1.4)	0.2 (0.2–0.3)	0.2 (0.1–0.3)	1.2 (0.4–1.9)
S2 (EC)		409 (96–713)	129 (98–160)	97 (47–146)	635 (241–1,020)	31 (7–55)	10 (7–12)	8 (4–11)	49 (18–78)
S3 (IT1)		35 (8–62)	9 (7–12)	7 (4–11)	52 (19–84)	4 (1–7)	1.1 (0.8–1.3)	0.8 (0.4–1.2)	6 (2–9)
S4 (IT2)		139 (33–240)	39 (30–49)	30 (14–45)	207 (77–333)	14 (3–24)	4 (3–5)	3 (1–5)	21 (8–34)
S5 (IT3)		296 (71–511)	88 (67–109)	66 (32–98)	450 (169–719)	28 (7–48)	8 (6–10)	6 (3–9)	42 (16–67)
S6 (AQG)		483 (117–829)	148 (113–184)	110 (53–165)	741 (283–1,177)	43 (10–73)	13 (10–16)	10 (5–15)	66 (25–104)

DALYs, disability-adjusted life years; LC, lung cancer; COPD, chronic obstructive pulmonary disease; DM, diabetes mellitus.

a The current scenario: S0, the scenario in 2019. The disease burden attributable to NO_2_ from gas cooking, secondhand smoking, and ambient were estimated in this scenario.

b Control scenarios: S1, smoking ban (SB); S2, cooking with electric stoves instead of gas stoves (EC); S3–6, restricting outdoor NO_2_ emissions to meet the World Health Organization (WHO) interim targets (ITs, IT1 = 40 μg/m^3^, IT2 = 30 μg/m^3^, IT3 = 20 μg/m^3^) and air quality guideline (AQG = 10 μg/m^3^). The reduction in disease burden attributable to NO_2_ was estimated in these scenarios.

The NO_2_-attributed DALYs and economic losses in urban areas in China in 2019 were divided according to the contribution of exposure from three sources of NO_2_: outdoor sources, cooking with gas appliances, and secondhand smoking. The sources contributing the most to the NO_2_-attributed burden of diseases were outdoor sources, associated with 1,004 (392–1,573) thousand DALYs and 86 (34–135) billion CNY economic losses, followed by cooking with gas appliances, associated with 657(258–1,030) thousand DALYs and 51 (20–79) billion CNY economic losses. Secondhand smoke contributed to less than 1% of NO_2_-attributed DALYs and economic losses, despite its much more hazardous effects for reasons well-known beyond NO_2_ production [35].

### 3.2 Attributable burden by city, sex, and age

**Fig. 2:**
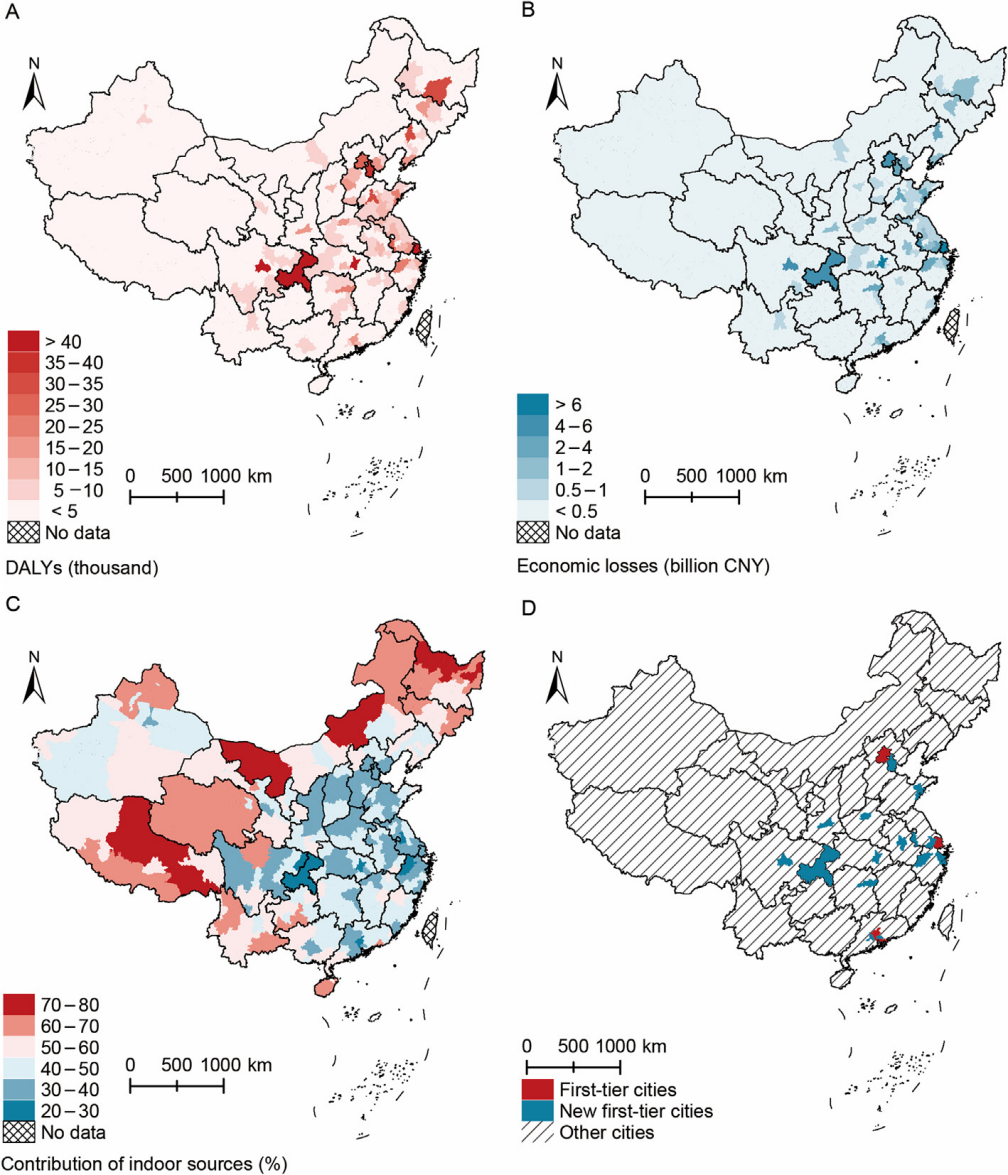
Disability-adjusted life years (DALYs) and economic losses attributable to NO_2_ in urban areas in 330 Chinese cities in 2019. (A) NO_2_-attributed DALYs. (B) NO_2_-attributed economic losses. (C) Percentage of NO_2_-attributed burden from indoor sources. (D) First-tier and new first-tier cities. Base map source: GS(2019)1822, http://bzdt.ch.mnr.gov.cn/index.html.

Among the 330 Chinese cities, the NO_2_-attributed burden of diseases was higher in urban areas in the first-tier and new first-tier Chinese cities (Fig. 2A and B), with the five highest burdens being observed in Chongqing, Shanghai, Wuhan, Chengdu, and Tianjin. This implies that demographic and economic factors may drive higher NO_2_-attributed DALYs and economic losses. Regions characterized by larger populations and robuster economic development tend to exhibit higher ambient NO_2_ concentrations, primarily due to factors such as intensified traffic and industrial emissions [36,37]. Consequently, these areas encompass both a larger population and higher NO_2_ exposure concentrations, thereby contributing to larger NO_2_-attributed disease burdens.

The percentage of the NO_2_-attributed burden from indoor sources (i.e., cooking with gas appliances and secondhand smoking) in northeast and northwest China was higher than that in other areas (Fig. 2C). This phenomenon may be attributed to the lifestyle in these areas, as supported by a survey involving over 100,000 individuals in China, which revealed that people in northeast and northwest China tend to spend more time indoors and seldom open their windows for ventilation [10,11]. Additionally, first-tier and new first-tier cities, where outdoor NO_2_ pollution is more severe, were mainly concentrated in central and southern China; therefore, indoor sources contribute less proportion to the NO_2_-attributed burden in these areas.

**Fig. 3:**
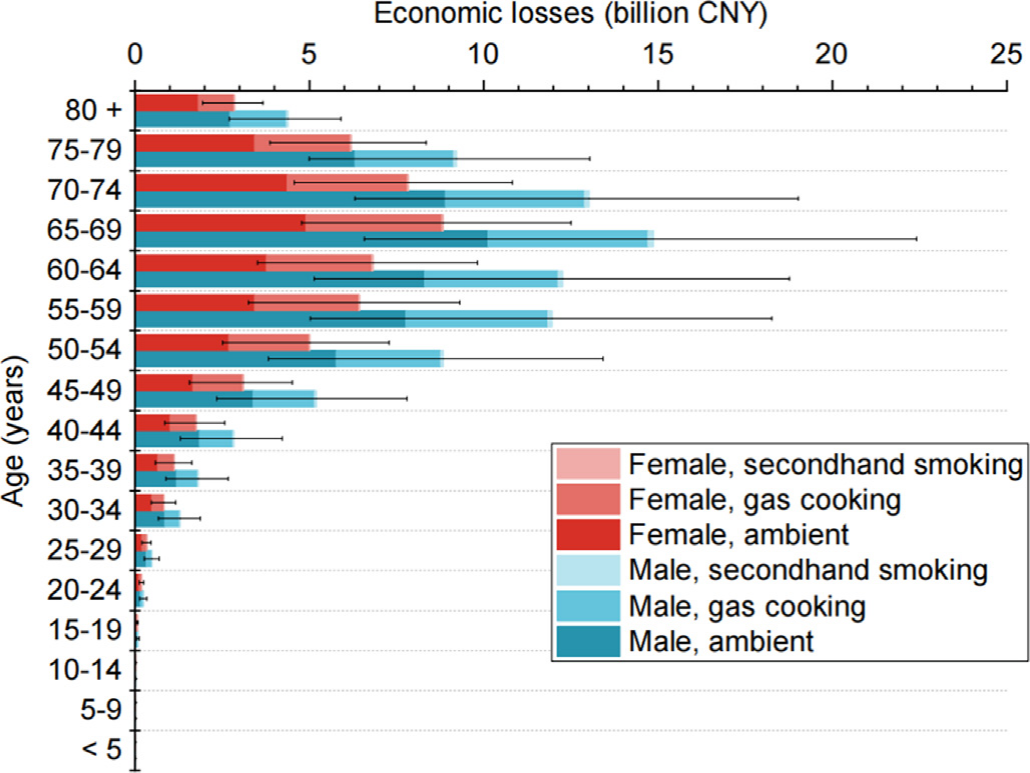
Economic losses attributable to NO_2_ by age and sex in urban China in 2019.

Our results showed sex and age disparities in the disease burden attributable to NO_2_ (Fig. 3 and Fig. S2). The NO_2_-attributed DALYs and economic losses in males [1,044 (387–1,655) thousand DALYs and 87 (32–137) billion CNY losses] were considerably higher than those in females [630 (268–969) thousand DALYs and 51 (22–79) billion CNY losses], as males are more likely to develop LC, COPD, and DM. The disease burden in the population under the age of 20 was very low, as they rarely develop LC, COPD, and DM. The proportion of NO_2_-attributed burden from indoor sources was higher in women aged 20 and above than in men in the same age group (Fig. S3), as women tend to engage in cooking activities more frequently than men, resulting in higher NO_2_ exposure concentrations from gas cooking [12].

### 3.3 Burden reduction by emission control

**Fig. 4:**
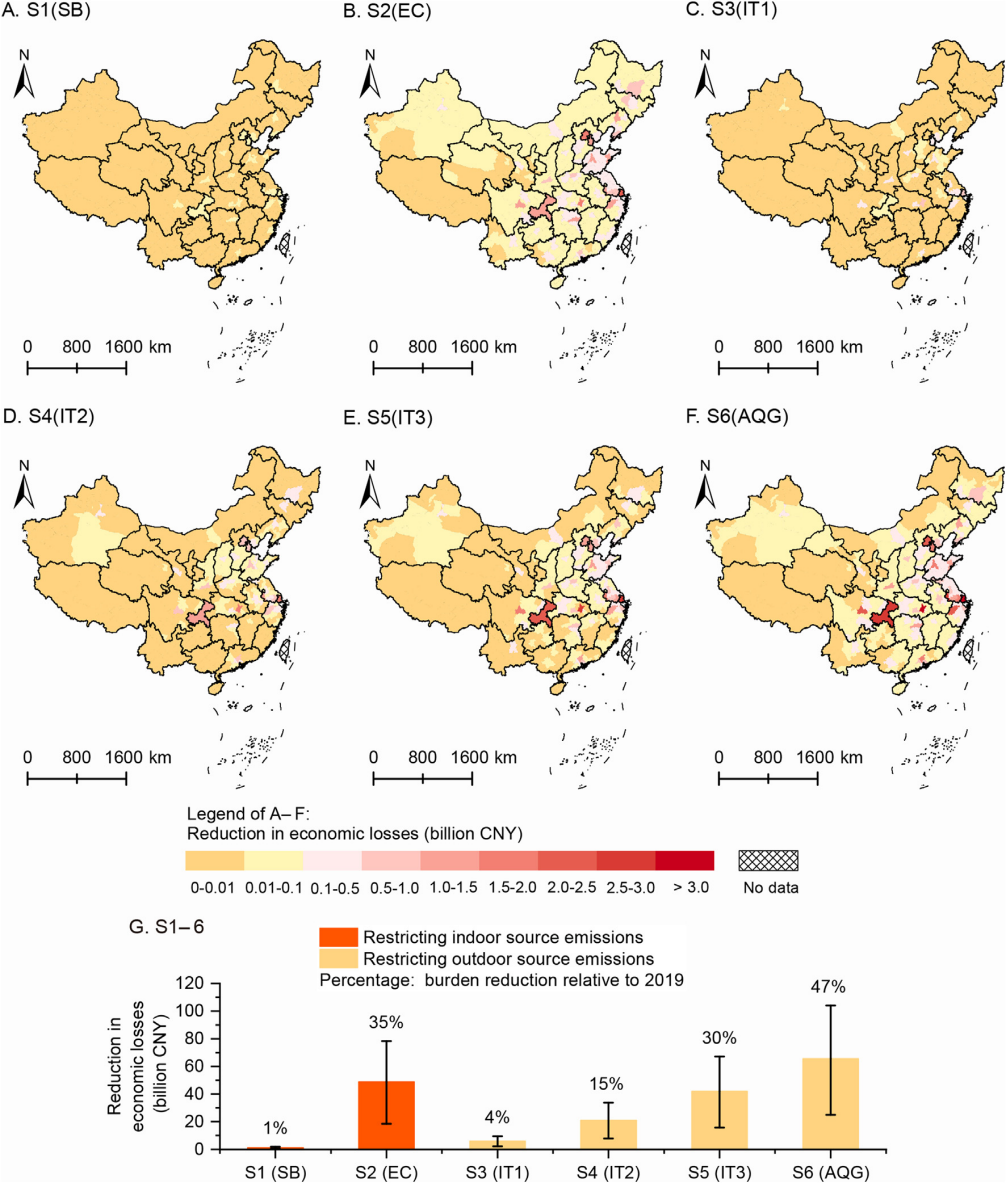
Reductions in economic losses attributable to NO_2_ in control scenarios S1–6 in 330 Chinese cities. (A) S1, smoking ban (SB); (B) S2, cooking with electric stoves instead of gas stoves (EC); (C–F) S3–6, restricting outdoor NO_2_ emissions to meet the World Health Organization (WHO) interim targets (ITs, IT1 = 40 μg/m^3^, IT2 = 30 μg/m^3^, IT3 = 20 μg/m^3^) and air quality guideline (AQG = 10 μg/m^3^); (G) the reduction in economic losses in urban China in control scenarios S1–6. Base map source: GS(2019)1822, http://bzdt.ch.mnr.gov.cn/index.html.

To assess the potential of NO_2_ mitigation measures to promote healthy living, we estimated the attributable burden reduction in six control scenarios (Table 1 and Fig. 4G). S6 (AQG) showed the largest reduction in NO_2_-attributed burden of disease, with a decrease of 741 (283–1,177) thousand DALYs and a 66 (25–104) billion CNY economic loss. The reduction in NO_2_-attributed burden in S2 (EC), with a reduction of 635 (241–1,020) thousand DALYs and 49 (18–78) billion CNY economic loss, is between that in the S5 (IT3) and S6 (AQG) scenarios. The reduction in NO_2_-attributed burden was negligible in S1 (SB).

**Fig. 5:**
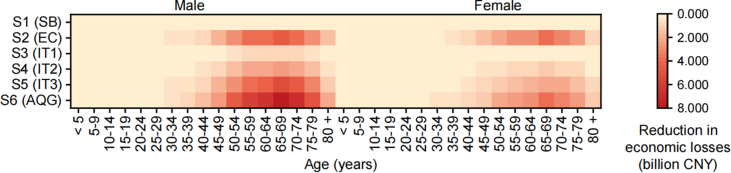
Reductions in economic losses attributable to NO_2_ in control scenarios S1–6 by age and sex. S1, smoking ban (SB); S2, cooking with electric stoves instead of gas stoves (EC); S3–6, restricting outdoor NO_2_ emissions to meet the World Health Organization (WHO) interim targets (ITs, IT1 = 40 μg/m^3^, IT2 = 30 μg/m^3^, IT3 = 20 μg/m^3^) and air quality guideline (AQG = 10 μg/m^3^).

Reducing indoor NO_2_ emissions showed relatively minor regional differences in reducing the disease burden across different regions (Figs. 4A, B, S4A, and S4B). However, reducing outdoor NO_2_ emissions to meet different targets (Figs. 4C–F and S4C–F) showed larger regional variation (S1 vs S3, S2 vs S6), with a significantly higher reduction in disease burden observed in first-tier and new first-tier cities. In 85% of the 330 cities in China, which had an urban population of 574 million, the reduction in NO_2_-attributed burden in S2 (EC) was higher than that in S5 (IT3), indicating that cooking with electric stoves rather than gas stoves is an effective way to protect people from NO_2_-attributed diseases. The other 15% of the 330 cities were mainly located in central and southern China, where the proportion of the NO_2_-attributed burden from indoor sources (from 27% to 39%, Fig. 2C) was lower than that in other areas (from 34% to 76%).

In terms of the reduction in NO_2_-attributed burden for different age groups (Figs. 5 and S5), these measures are most effective in reducing the disease burden attributable to NO_2_ in the population aged 20 and above, with a larger reduction seen in males than in females in the same scenario. Our comparisons of the different control measures revealed that, in S2 (EC), the reduction in economic losses for males aged 20 and above was between that in S4 (IT2) and S5 (IT3), whereas, for females aged 20 and above, the reduction in economic losses in S2 (EC) was larger than that in S6 (AQG). The sex differences indicated that, particularly for individuals who regularly cook at home (which is more common among females than males in China), switching from gas to electric stoves may be a more effective measure for reducing the disease burden attributable to NO_2_ than reducing outdoor NO_2_ emissions.

## 4 Discussions

NO_2_ pollution has severe implications for public health in the urban areas of China. In 2019, NO_2_ pollution was associated with millions of DALYs owing to LC, COPD, and DM, resulting in economic losses equivalent to a thousandth of China's GDP in the same year. Both indoor and outdoor sources of NO_2_ contributed significantly to the disease burden, highlighting the urgent need for further control measures to reduce NO_2_ emissions from both sources. Apart from the current measures aimed at reducing atmospheric NO_2_, switching from gas stoves to electric stoves in homes is a crucial measure in mitigating the burden of NO_2_-attributed diseases, with a potential 35% reduction in the related economic losses in China. To the best of our knowledge, this study is the first to quantify the disease burden and economic losses associated with NO_2_-attributed LC, COPD, and DM, as well as to differentiate between indoor and outdoor sources of NO_2_ pollution.

NO_2_ is widely acknowledged as an irritant gas that can trigger respiratory illnesses upon inhalation. Global studies have estimated 4.0 (1.8–5.2) million [24], 3.5 (2.1–6.0) million [25], and 1.9 (0.9–2.8) million [9] pediatric asthma cases worldwide in 2015, 2015, and 2019, respectively, attributed to atmospheric NO_2_ pollution, with China experiencing the highest disease burden. In recent years, an increasing number of national- and city-level studies have explored other health outcomes, including respiratory diseases such as COPD and LC, cardiovascular diseases [19,20], metabolic diseases such as DM, and associated mortality and DALY loss [22,38]. According to studies conducted in China, hundreds of thousands of premature deaths are attributed to ambient NO_2_ pollution each year. Qi et al. reported that between 2013 and 2020, an estimated annual death of 279 (189–366) to 339 (231–442) thousand was attributed to atmospheric NO_2_ pollution from non-accidental diseases, including cardiovascular and respiratory disease [19]. Xue et al. reported 315 (307–319) thousand premature deaths attributed to atmospheric NO_2_ in 2013 and 250 (242–254) thousand in 2020 [39]. Li et al. Reported that long-term exposure to atmospheric NO_2_ was associated with 285 (144–558) thousand premature deaths in 2019 [40]. This study found that, in China's urban areas, where ambient NO_2_ pollution is severe compared to other areas in China, 1,004 (392–1,573) thousand DALYs from LC, COPD, and DM were attributed to atmospheric NO_2_ pollution in 2019. Similar results were reported in developed countries and regions such as Europe and the United States, suggesting that atmospheric NO_2_ pollution was associated with non-communicable disease morbidity and mortality [23,38,41,42]. Remarkably, previous studies have not explored the disease burden attributed to NO_2_ from indoor sources, except for one study that estimated 166 (91–223) thousand NO_2_-attributed pediatric asthma cases [27]. A key novelty of this study is the comprehensive estimation of richer health outcomes across all age groups, assessing an additional 671 (263–1,051) thousand DALYs attributable to NO_2_ from indoor sources, particularly household gas consumption. As gas is usually considered clean household energy [3], the use of gas appliances is prevalent in both developed and developing countries, with some rural areas transitioning from solid fuel to gas as their household energy [4]. This indicates that a large number of people worldwide are exposed to NO_2_ generated from household gas consumption, leading to a significant disease burden. Emerging evidence of the health outcomes associated with NO_2_ pollution underscores the importance of raising awareness and promoting effective intervention on a global scale, particularly in terms of increasing the awareness of gas as a polluting household energy.

Current control measures for outdoor NO_2_ pollution mainly target the treatment of exhaust gases from industries, power plants, and vehicles [[43], [44], [45], [46]], as well as the development of zero-carbon technologies such as zero-carbon electricity and zero-emission vehicles [47]. Effective mitigation of NO_2_ pollution requires tailored interventions that consider its characteristics as a typical urban air pollutant associated with both indoor and outdoor fossil energy consumption, including transportation, power industries, and household gas consumption. Consequently, the transformation of urban areas from fossil fuels to non-fossil fuels in these three aspects is necessary to alleviate NO_2_ pollution. The development of zero-carbon technologies is in line with global efforts to combat climate change [48], as evidenced by the commitment of 65 countries and major sub-national economies to achieve net-zero greenhouse gas emissions by 2050 at the 2019 Climate Action Summit. Compared to the decarbonization efforts made by various countries in the transportation and power sectors, which were estimated to reduce atmospheric NO_x_ concentrations by 19%–80% in China [49,50] and by 3%–60% in Europe [51] at the same time, household gas consumption has received little attention in the energy sector owing to its low energy consumption share. However, switching from gas stoves to electric stoves can significantly reduce the disease burden associated with NO_2_ from a public health perspective. Thus, it is essential to raise public awareness regarding the health risks associated with gas cooking, develop convenient electric cooking technologies, and implement policies to encourage urban residents to switch to electric stoves. This study suggests that indoor smoking bans have a limited effect on reducing the disease burden attributable to NO_2_, owing to the low contribution of secondhand smoke to NO_2_ exposure concentrations. Nonetheless, promoting indoor smoking bans is essential because smoking emits other air pollutants that affect both smokers and secondhand smokers [35].

This study has the following limitations. First, in the process of estimating NO_2_ exposure, we derived the NO_2_ emission rate during gas combustion from only one study conducted in the United Kingdom, as this study provides detailed value for our estimation. We also found the latest research conducted in the United States [6] and China [52,53] reporting similar NO_2_ emission rates during gas combustion, demonstrating a negligible variance (less than 10%) when compared to the emission rate employed in our study. Second, this study only focused on NO_2_ produced from gas combustion during cooking, without considering other household gas combustion processes, such as using wall-mounted gas boilers. Wall-mounted gas boilers used in Chinese households are typically equipped with exhaust pipes, resulting in the NO_2_ produced from gas combustion being released outdoors and a minimal contribution to indoor NO_2_ concentrations [54]. Third, the concentration–response functions obtained in our previous publication were based on epidemiological studies that combined outdoor and indoor environments [31]. This may not precisely correspond to the evaluation of the disease burden of NO_2_ exposure from indoor and outdoor sources. However, because the target compound was the same as NO_2_, we believe that this would not introduce a large bias. In addition, we set the maximum relative risk (*RR*) constrained to the maximum concentration. This may have led to an underestimated evaluation of concentrations higher than the maximum. Epidemiological studies involving *RR* estimations beyond the maximum concentration are required. Fourth, we applied the concentration–response function from a global meta-analysis to estimate the NO_2_-attributed burden of diseases in urban areas in China. Importantly, about half of the data in the meta-analysis originated from China, which significantly contributed to reducing uncertainty when applying these findings to an urban Chinese population. However, it's essential to acknowledge that there may still be some residual uncertainty associated with potential population differences. Fifth, our study focused exclusively on three specific health outcomes (LC, COPD, and DM), even though NO_2_ exposure has been associated with a broader spectrum of diseases, including pediatric asthma and cardiovascular diseases. However, for these additional health outcomes, either our referenced meta-analysis did not consistently reveal significant associations with NO_2_ exposure, as observed with cardiovascular diseases [31], or their contribution to DALYs was relatively minor, and previous research had already estimated their NO_2_-attributed disease burden, such as pediatric asthma [27]. Our study may be characterized as providing a relatively conservative estimate of the DALYs associated with NO_2_ exposure, but does not introduce significant bias into the assessment. Sixth, the motivation for using the human capital approach, assuming that one DALY is equal to one GDP per capita losses, is to provide a measure that is easily relatable to policymakers and commonly used in economic assessments of disease burden. Different age groups may have varying contributions to society and different economic impacts, which are not considered in this analysis. Setting reasonable productivity coefficients for different age groups is difficult, as values can be challenging to ascertain and can vary significantly across regions and populations. Several reviews on numerous studies revealed that one DALY was equal to one GDP per capita applicable [33,55], which reinforced the rationale for employing this approach.

In summary, by quantifying the disease burden associated with indoor and outdoor sources of NO_2_ and comparing the effectiveness of various control measures, this study identified the important public health impacts of NO_2_ from outdoor sources and household gas cooking. We emphasize that gas is not a clean household cooking energy and recommend switching from gas stoves to electric stoves to promote public health.

## CRediT authorship contribution statement

Y.H. designed the study, planned the analysis, collected data, performed the model analysis, analyzed the simulation results, interpreted the results, validated and completed all figures, and drafted the manuscript. Y.W. collected data. Z.H.Z. provided data, drafted and commented on the manuscript. B.Z. coordinated and supervised the project, designed the study, planned the analysis, analyzed the simulation results, interpreted the results, and drafted the manuscript.

## Declaration of competing interests

The authors declare no competing interests.
