# Supplementary material for: Reconsidering gas as clean energy: Switching to electricity for household cooking to reduce NO2-attributed disease burden
Source: Eco Environ Health. 2023 Nov 17;3(2):174–82. doi: 10.1016/j.eehl.2023.10.003 (PMC11021829; doi:10.1016/j.eehl.2023.10.003)
Supplement: Multimedia component 1 [file mmc1.pdf]

Supplementary materials for

Reconsidering Gas as Clean Energy: Switching to Electricity for Household Cooking to Reduce NO_2-attributed Disease Burden

Ying Hu[a, †], *Ye Wang*[a, †], *Zhuohui Zhao*[b,c,d], *Bin Zhao*[a,e,*]

[a] Department of Building Science, School of Architecture, Tsinghua University, Beijing, 100084, China.
[b] School of Public Health, Fudan University, Shanghai, China.
[c] Key Laboratory of Public Health Safety of the Ministry of Education, NHC Key Laboratory of Health Technology Assessment, Fudan University, Shanghai, China.
[d] Shanghai Typhoon Institute/CMA, Shanghai Key Laboratory of Meteorology and Health, IRDR International Center of Excellence on Risk Interconnectivity and Governance on Weather/Climate Extremes Impact and Public Health, WMO/IGAC MAP-AQ Asian Office Shanghai, Fudan University, Shanghai, China
[e] Beijing Key Laboratory of Indoor Air Quality Evaluation and Control, Tsinghua University, Beijing, 100084, China.

[†] Ying Hu and Ye Wang contributed equally to this work.

* Corresponding author:

Bin Zhao, Department of Building Science, School of Architecture, Tsinghua University,

Beijing 100084, China. Email: binzhao@tsinghua.edu.cn

Supplementary experimental procedures

Source-specific exposure model 3

DALY rates and population in China 3

Robustness of Monte Carlo simulation 4

Supplementary figures

Figure S1 Framework of source-specific exposure model. 5

Figure S2 DALYs attributable to NO_2 by age and sex in 2019. 6

Figure S3 Proportion of economic losses attributable to NO_2 from different sources. 7

Figure S4 Reductions in DALYs in 330 Chinese cities in S1~6. 8

Figure S5 Reductions in DALYs in S1~6 by age and sexes 9

Figure S6 NO_2 exposure concentration in urban areas in 330 Chinese cities. 10

Figure S7 NO_2 exposure concentration in urban areas by age and sex 11

Supplementary tables

Table S1 Validation of the source-specific exposure model 12

Table S2 NO_2 exposure concentration from outdoor sources ($C_{ambient}$), gas cooking ($C_{cooking}$), and second-hand smoke (C_{SHS}) after restrictions on NO_2 emissions. 13

Table S3 DALY rates (per 100 000) for all ages and both sexes in 31 Chinese provinces. 14

Table S4 DALY rates (per 100 000) for different age and sexes groups in China in 2019. 15

Table S5 Population (unit: thousand) and per capita Gross Domestic Product per year (unit: CNY) in urban areas of 330 Chinese city in 2019. 16

Table S6 Proportion for populations with different ages and genders in 31 Chinese provinces. ... 19

Table S7 Exposure factor (f_{exp}) of NO_2 in 31 provinces in China. 20

Supplementary experimental procedures

Source-specific exposure model

We have developed a source-specific exposure model[1] to simulate NO_2 exposure concentration from various sources (**Figure S1**). The model first established an equation to calculate the indoor concentration of NO_2 based on the conservation of mass combined with indoor dynamics of NO_2. It then separated the indoor concentration of NO_2 from outdoor sources ("aC_{out}") and indoor sources ("*S/V*"), denoted as $C_{in,o}$ and $C_{in,i}$ in **Figure S1**, respectively. Human activities both indoors and outdoors were taken into account to calculate the NO_2 exposure concentration from outdoor ("$C_{exp,o}$") and indoor ("$C_{exp,i}$") sources. A two-stage Monte Carlo approach was applied in the model to obtain the variability distribution of exposure. We validated the model by comparing the modelled concentrations with available field studies, ensuring the credibility of the modelled exposure (**Table S1**).

The input parameters used in this model to estimate NO_2 exposure concentration in urban China in 2019 included the outdoor concentration of NO_2 from 1,497 monitoring stations in 333 cities in China, the emission rate of NO_2 from gas cooking and smoking, the surface removal rate of NO_2 indoors, air change rates when windows were open, closed, and when operating range hoods, the volume of air in residences, and habits of cooking, smoking, ventilation, and outdoor activities. These parameters were detailed in Table 1 of our previous study[1], and here we only describe one of the key parameters — estimation of NO_2 emissions from gas cooking. This estimation relies on a comprehensive dataset of both the rate of NO_2 generation from gas combustion and the cooking habits of residences in urban areas in China. The rate of NO_2 generation from gas combustion was from a study conducted in the United Kingdom [2]. To adapt this data to the Chinese context, we adjusted based on household gas consumption and total cooking duration specific to China [3]. Regarding cooking habits, we conducted an extensive survey spanning all 31 provinces in China, which yielded a substantial dataset comprising 1103 valid responses [1]. This survey collected a wealth of information related to gas usage for cooking, encompassing parameters such as cooking frequency, time periods for cooking activities, preferred cooking methods, and other factors that could influence indoor NO_2 concentrations during gas cooking, such as ventilation. Using this comprehensive dataset, we were able to simulate how individuals used gas for cooking daily, including when they cooked and at what emission rates. These data served as crucial input parameters for the model presented in **Figure S1**, forming foundation for NO_2 exposure concentration estimation.

DALY rates and population in China

The equation used to calculate the DALY rate for a specific age group and sex in a specific province in China in 2019 was as follows:

$$DALY\ rate_{province,age,sex} = DALY\ rate_{province,all\ ages\ and\ sexes} \times \frac{DALY\ rate_{China,age,sex}}{DALY\ rate_{China,all\ ages\ and\ sexes}} \qquad (S1)$$

The DALY rates for all ages and both sexes in 31 Chinese provinces were obtained from a dataset provided by *National Centre for Chronic and Noncommunicable Disease Control and Prevention* (**Table S3**)[4]. The DALY rates for people of different age and sexes groups were from the Global Burden of Disease Study 2019 (GBD 2019) (**Table S4**)[5].

The age and sex-specific population size in urban areas in 31 Chinese provinces in 2019 was calculated based on the urban population of each city in 2019[6] (**Table S5**) and the age and gender composition of the population of each province[7] (**Table S6**).

Robustness of Monte Carlo simulation

The model produces different results each time it runs a given number of repetitions. The standard deviation of these results is called Monte-Carlo Error (MCE) [8]. We defined SD as the minimum value of the standard deviation of the target output in 250 simulations. The MCE to SD ratio (MCE/SD) is the measurement of the uncertainty of Monte Carlo simulation compared with the variability of the distribution of the simulated results [9]. In our previous study, we calculated MCE/SD values for the variability stage to be less than 5%[1], and, in this study, MCE/SD values for the uncertainty stage were also less than 5%, indicating that 2000*1000 iterations were adequate. [10]

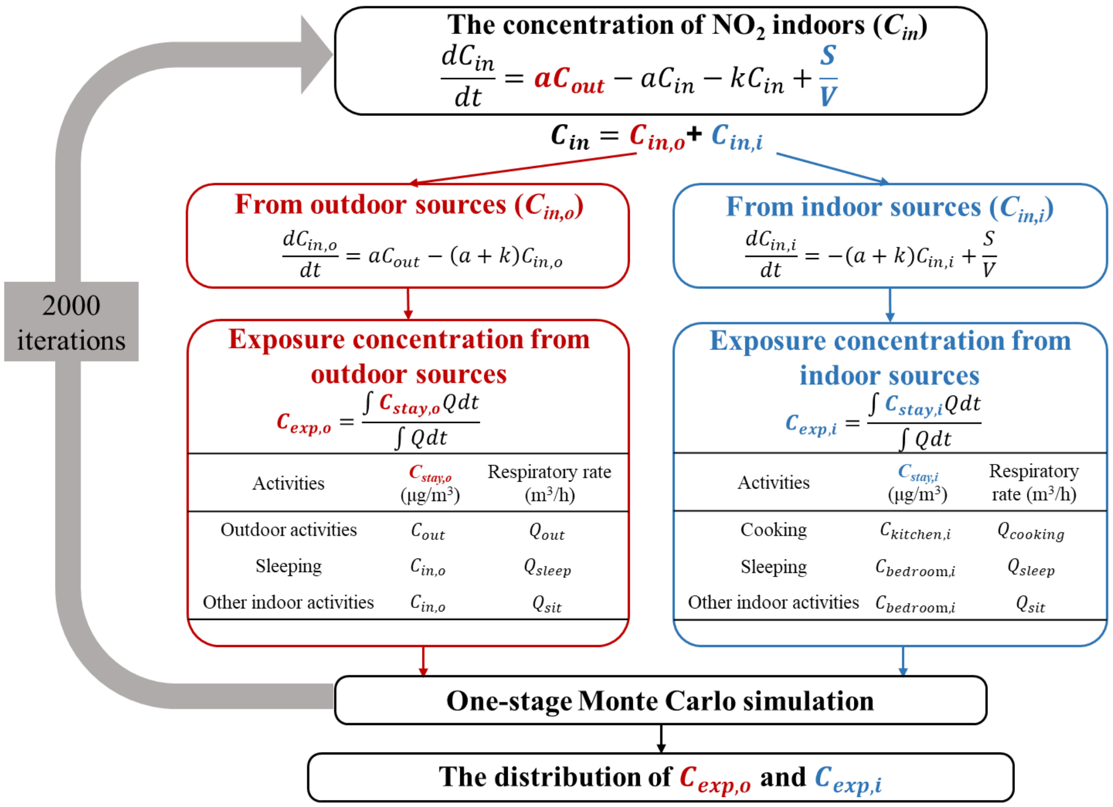

- *t (h): time;*
- *a (h^{-1}): air exchange rate with the outdoor environment, under three conditions including closed window, open window, and using the range hood;*
- *C_{out} (μg/m³): the concentration of NO_2 outdoors;*
- *k (h^{-1}): surface removal rate of NO_2 indoors;*
- *S (μg/h): emission rate of NO_2, including gas cooking and smoking;*
- *V (m³): the volume of indoor air.*

- *$C_{stay,o}$ (μg/m³): NO_2 exposure concentration from outdoor sources where the person is staying;*
- *$C_{stay,i}$ (μg/m³): NO_2 exposure concentration from indoor sources where the person is staying;*

Figure S1 Framework of source-specific exposure model.

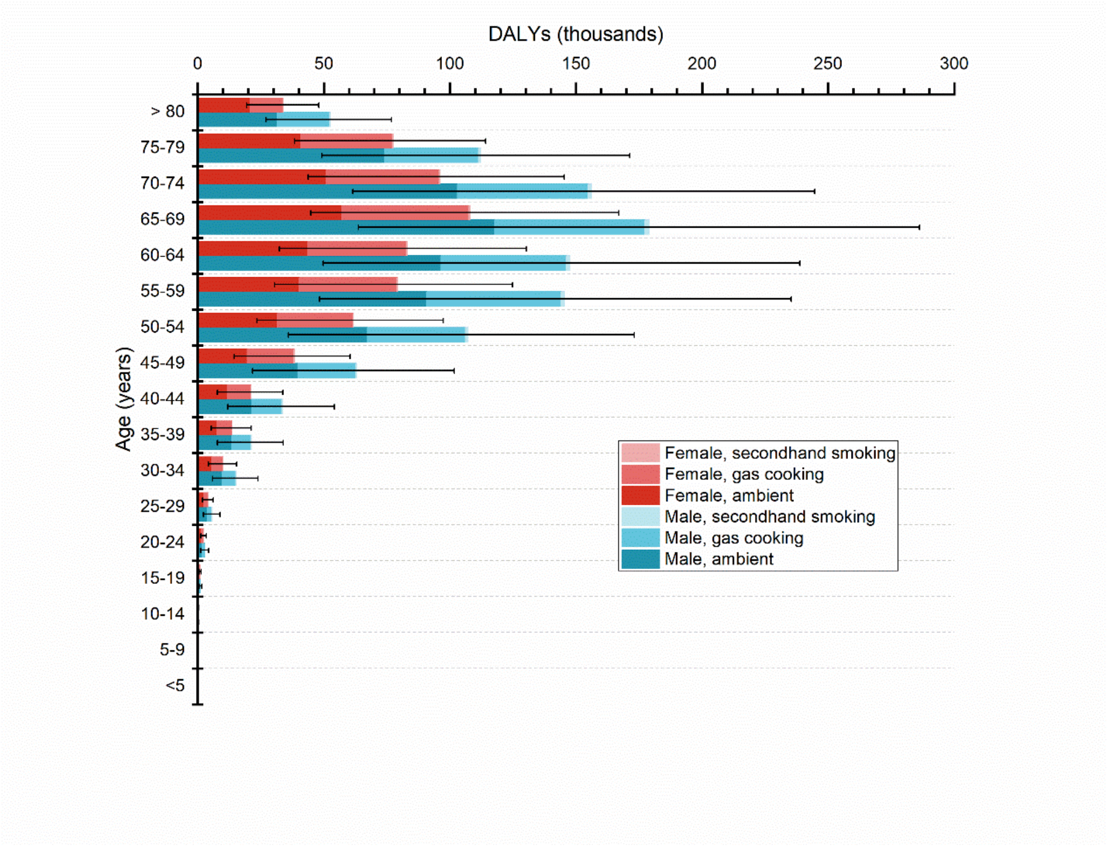

Figure S2 DALYs attributable to NO_2 by age and sex in 2019.

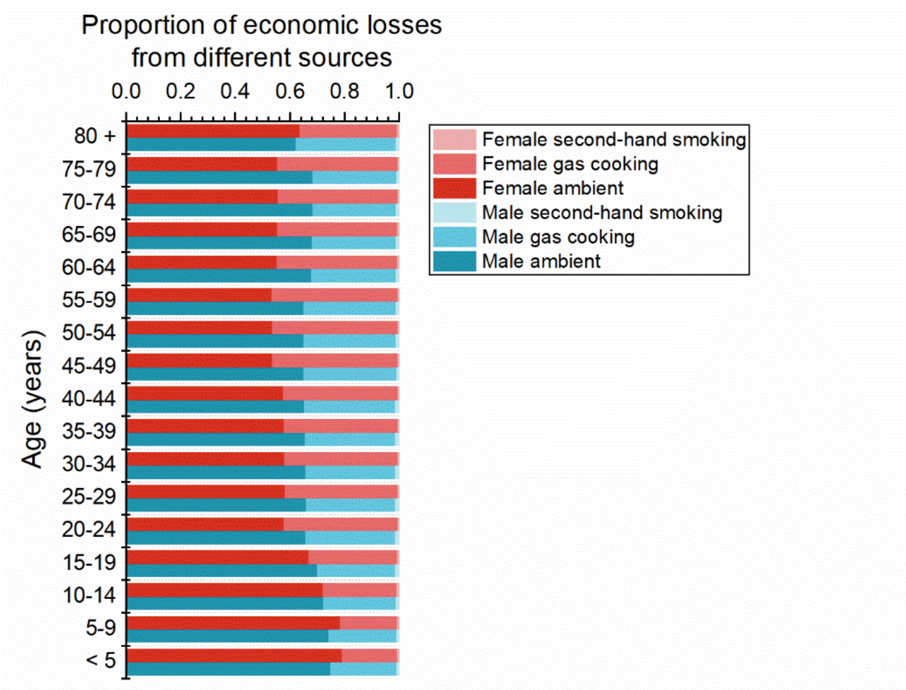

Figure S3 Proportion of economic losses attributable to NO_2 from different sources in 2019.

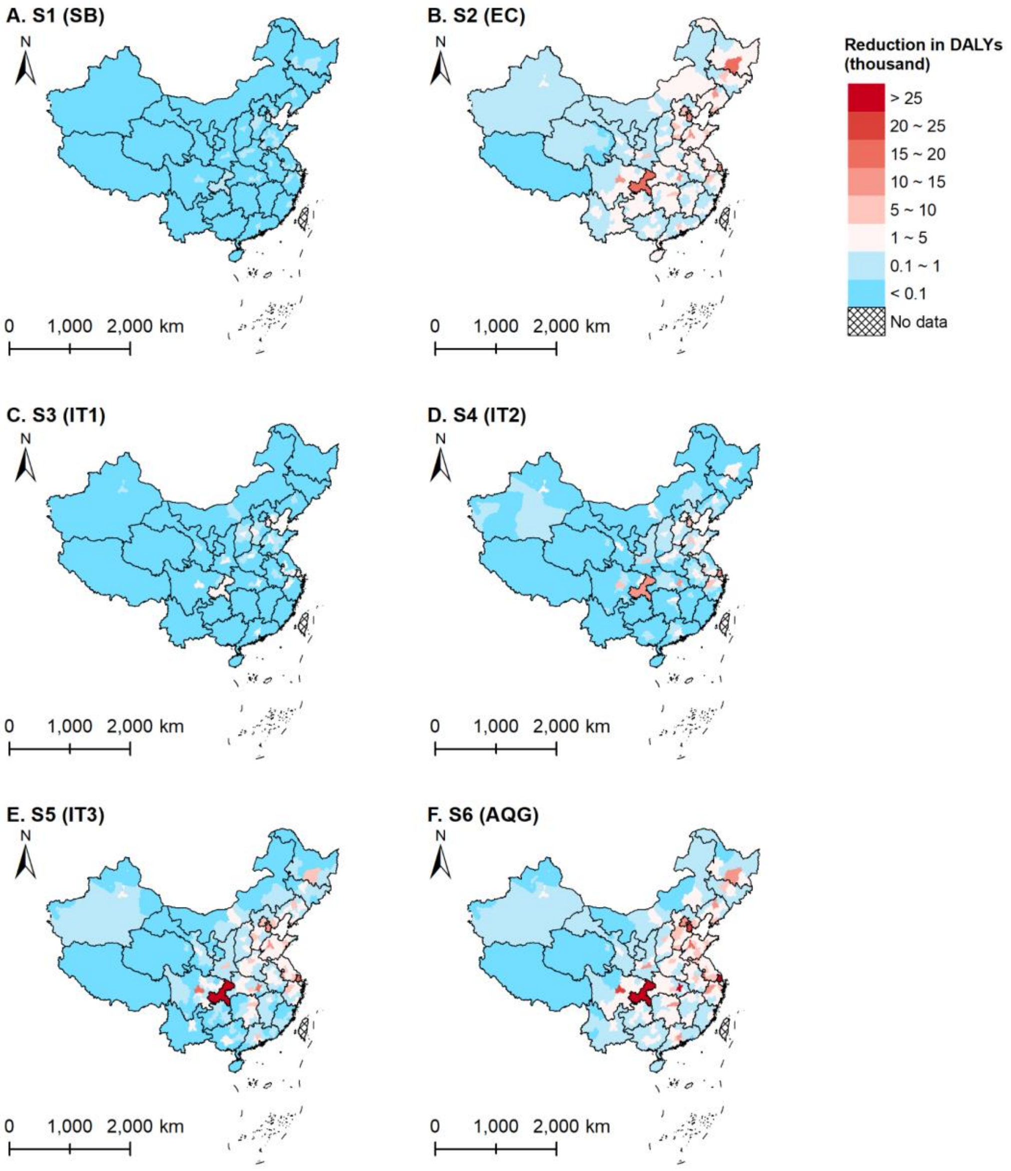

Figure S4 Reductions in DALYs in 330 Chinese cities in S1~6. A. S1, smoking ban (SB); B. S2, cooking with electric stoves instead of gas stoves (EC); C–F. S3–6, restricting outdoor NO_2 emissions to meet the World Health Organization (WHO) interim targets (IT, IT1=40 μg/m^3, IT2=30 μg/m^3, IT3=20 μg/m^3) and air quality guideline (AQG =10 μg/m^3). Base map source: GS(2019)1822, http://bzdt.ch.mnr.gov.cn/index.html.

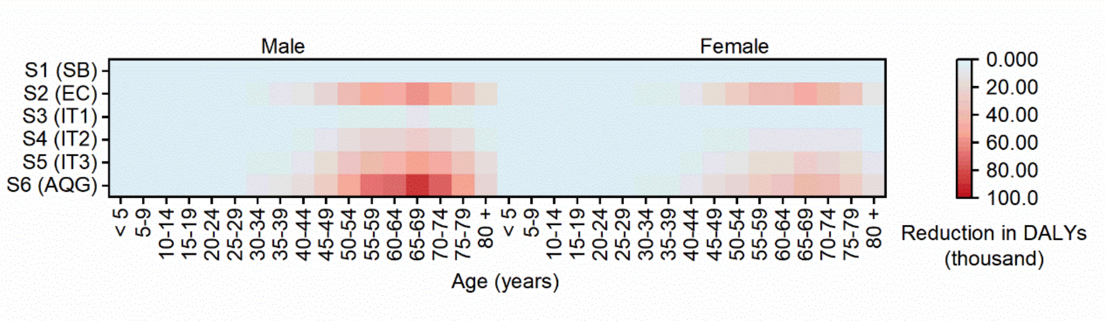

Figure S5 Reductions in DALYs in S1~6 by age and sexes. S1, smoking ban (SB); S2, cooking with electric stoves instead of gas stoves (EC); S3–6, restricting outdoor NO_2 emissions to meet the World Health Organization (WHO) interim targets (ITs, IT1=40 μg/m^3, IT2=30 μg/m^3, IT3=20 μg/m^3) and air quality guideline (AQG =10 μg/m^3).

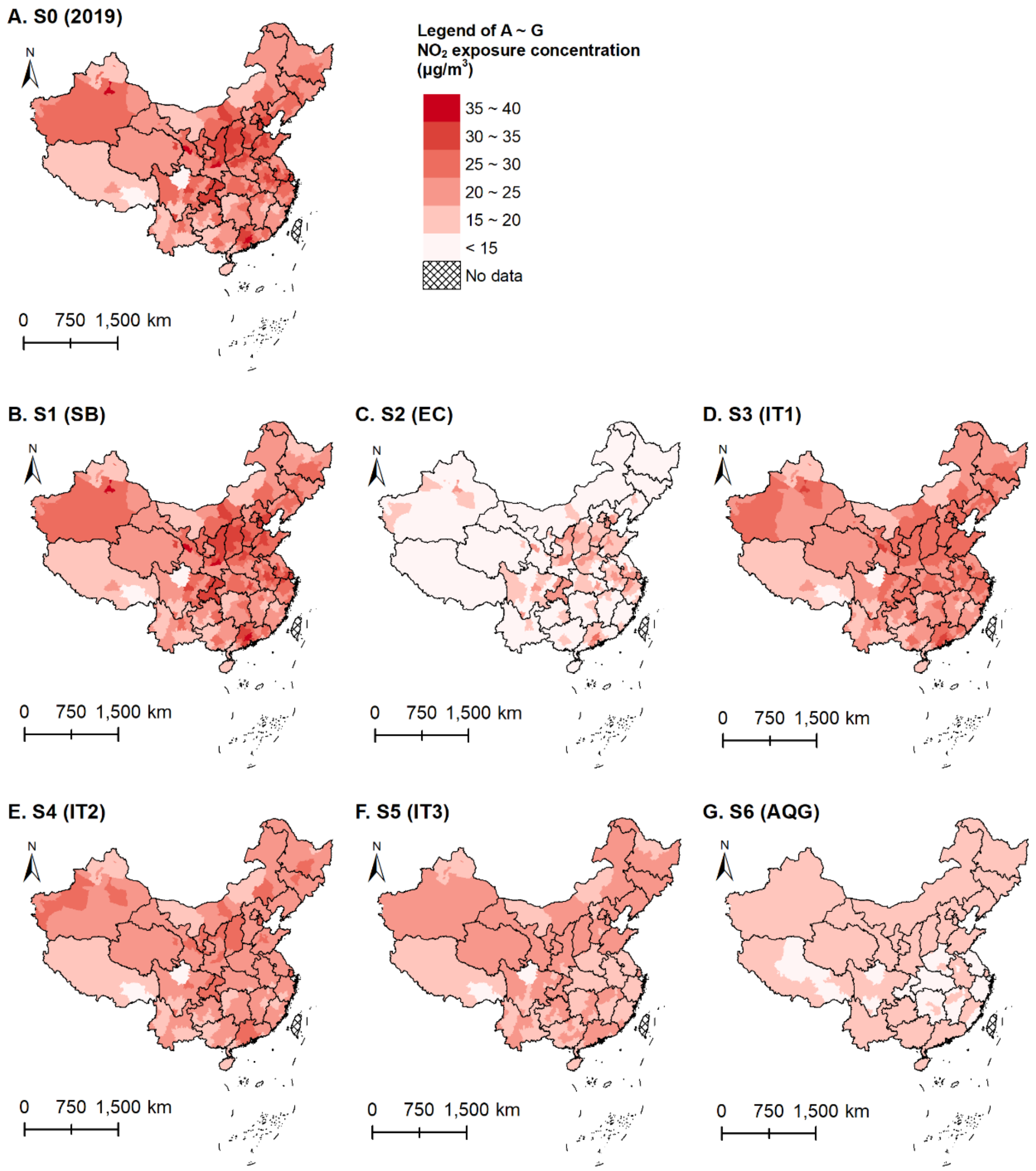

Figure S6 NO_2 exposure concentration in urban areas in 330 Chinese cities. A. S0, in 2019; B. S1, smoking ban (SB); C. S2, cooking with electric stoves instead of gas stoves (EC); D–G. S3–6, restricting outdoor NO_2 emissions to meet the World Health Organization (WHO) interim targets (ITs, IT1=40 μg/m^3, IT2=30 μg/m^3, IT3=20 μg/m^3) and air quality guideline (AQG =10 μg/m^3). Base map source: GS(2019)1822, http://bzdt.ch.mnr.gov.cn/index.html.

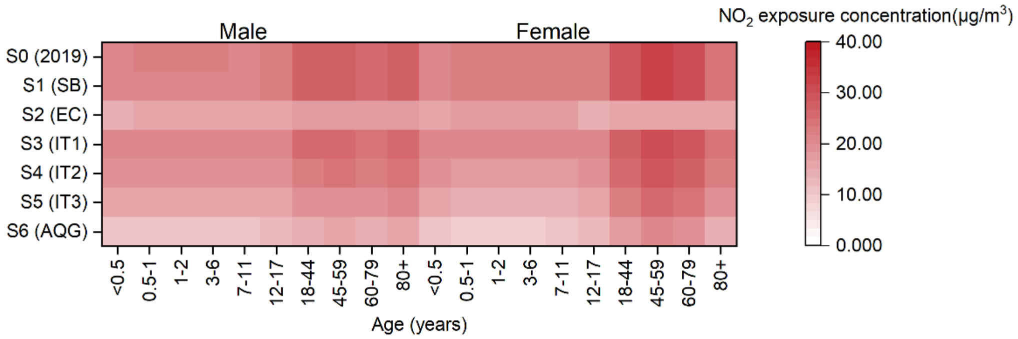

Figure S7 NO_2 exposure concentration in urban areas by age and sex. S0, in 2019; S1, smoking ban (SB); S2, cooking with electric stoves instead of gas stoves (EC); S3–6, restricting outdoor NO_2 emissions to meet the World Health Organization (WHO) interim targets (ITs, IT1=40 μg/m^3, IT2=30 μg/m^3, IT3=20 μg/m^3) and air quality guidelines (AQG =10 μg/m^3).

Table S1 Validation of the source-specific exposure model.

City		Modeled Mean (95% CI)	Measured Mean (range)	Relative errors of mean values	References
Dalian	Kitchen	38.5 (32.2, 50.0)	36.3 (5.2, 141)	6%	[11]
Dalian	Bedroom	23.5 (16.3, 39.2)	18.7 (2.5, 49.1)	26%	
Shanghai	Exposure	21.0 (11.2, 37.9)	27.4 (1.14, 123.21)	23%	[12]
Qingdao	Heating season	56.2 (14.5, 117.4)	51.0 (9.5, 197)	10%	[13]
Shenzhen	Bedroom and living room	25.7 (15.0, 38.4)	28 (14, 35)	8%	[14]
Xian and Lanzhou	Indoor	27.5 (12.1, 44.2)	34 (10, 125)	20%	[15]

Table S2 NO_2 exposure concentration from outdoor sources ($C_{ambient}$), gas cooking ($C_{cooking}$), and second-hand smoke (C_{SHS}) in seven scenarios.

Scenario	$C_{ambient}$	$C_{cooking}$	C_{SHS}
S0. Current scenario	$C_{ambient}$ in 2019	$C_{cooking}$ in 2019	C_{SHS} in 2019
S1. SB[a]	$C_{ambient}$ in 2019	$C_{cooking}$ in 2019	0
S2. EC[b]	$C_{ambient}$ in 2019	0	C_{SHS} in 2019
S3. IT1[c]	Equation (1), *Target*=40μg/m^3	$C_{cooking}$ in 2019	C_{SHS} in 2019
S4. IT2	Equation (1), *Target*=30μg/m^3	$C_{cooking}$ in 2019	C_{SHS} in 2019
S5. IT3	Equation (1), *Target*=20μg/m^3	$C_{cooking}$ in 2019	C_{SHS} in 2019
S6. AQG[d]	Equation (1), *Target*=10μg/m^3	$C_{cooking}$ in 2019	C_{SHS} in 2019

[a] SB, smoking ban: no people smoking indoors;

[b] EC, using electric stoves for cooking: all residents using electric stoves for cooking in Chinese urban areas;

[c] IT, the outdoor air meets the World Health Organization interim target for NO_2, IT1 = 40 μg/m^3, IT2 = 30 μg/m^3, IT3 = 20 μg/m^3;

[d] AQG, the outdoor air meets the World Health Organization Air Quality Guideline for NO_2, AQG = 10 μg/m^3.

Table S3 DALY rates (per 100 000) for all ages and both sexes in 31 Chinese provinces.

Province	Lung cancer	Chronic obstructive pulmonary disease	Diabetes mellitus
Beijing	703.88 (576.67, 832.94)	640.3 (553.97, 741.35)	753.51 (552.01, 1008.76)
Tianjin	1266.04 (1067.31, 1517.41)	785.43 (679.74, 931.71)	955.5 (720.67, 1241.18)
Hebei	977.17 (809.69, 1156.84)	1060.89 (926.5, 1270.68)	787.09 (606.2, 1008.15)
Shanxi	822.13 (654.64, 1002.84)	890 (760.03, 1086.48)	700.42 (513.89, 927.34)
Inner Mongolia	953.81 (804.11, 1127.58)	1249.18 (1103.15, 1415.49)	715.36 (523.55, 940.96)
Liaoning	1780.95 (1480.92, 2118.38)	1077.75 (918.12, 1402.65)	1106.65 (869.58, 1381.27)
Jilin	1207.93 (980.48, 1425.64)	806.07 (690.95, 1075.56)	859.27 (645.74, 1112.72)
Heilongjiang	1918.39 (1594.24, 2275.08)	1185.23 (1026.83, 1538.41)	889.79 (674.6, 1148.78)
Shanghai	739.38 (609.17, 897.39)	855.4 (759.19, 970.68)	755.74 (571.9, 980.05)
Jiangsu	1238.83 (1030.21, 1469.82)	1523.61 (1347.48, 1707.97)	789.51 (606.97, 1008.21)
Zhejiang	1053.64 (866.82, 1265.26)	1065.22 (945.86, 1191.84)	596.05 (445.9, 773.97)
Anhui	1119.86 (941.61, 1337.13)	1318.96 (1167.06, 1544.25)	714.46 (552.07, 907.2)
Fujian	955.45 (789.38, 1156.03)	945.46 (834.15, 1065.54)	662.53 (511.44, 857.93)
Jiangxi	878.51 (748.57, 1035.75)	1429.64 (1281.28, 1594.34)	547.45 (418.48, 695.28)
Shandong	1470.82 (1227.17, 1737.88)	1372.8 (1204.67, 1578.51)	653.77 (494.15, 849.63)
Henan	942.21 (786.97, 1113.63)	1148.76 (1010.35, 1420.88)	685.46 (538.42, 861.57)
Hubei	1357.37 (1135, 1610.69)	1733.77 (1529.56, 1947.52)	637.96 (500.33, 815.58)
Hunan	1318.57 (1104.37, 1590.24)	2061.94 (1835.78, 2323.72)	773.44 (599.54, 971.47)
Guangdong	762.98 (636.87, 907.04)	1003.65 (897.85, 1125.88)	647.47 (477.01, 852.46)
Guangxi	857.46 (719.16, 1028.1)	1415.69 (1260.28, 1590.04)	625.66 (485.2, 788.19)
Hainan	623.6 (501.73, 782.47)	1034.36 (894.54, 1201.16)	768.21 (575.36, 994.11)
Chongqing	1480.75 (1186.99, 1848.17)	2662.15 (2291.46, 3099.95)	692.22 (526.3, 882.58)
Sichuan	1508.43 (1226.91, 1778.62)	2960.45 (2558.43, 3340.52)	722.65 (566.07, 905.75)
Guizhou	784.48 (644.17, 938.3)	2205.06 (1948.27, 2461.48)	647.15 (505.03, 814.52)
Yunnan	684.52 (576.6, 818.81)	2120.01 (1863.31, 2385.49)	615.36 (482.28, 770.09)
Tibet	118.42 (97.54, 142.94)	1579.09 (1359.28, 1820.95)	393.84 (300.18, 509.06)
Shaanxi	728.89 (582.97, 905.15)	1044.68 (905, 1230.96)	601.81 (461.99, 769.85)
Gansu	542.65 (451.97, 646.94)	2177.61 (1900.26, 2450.54)	641.46 (503.25, 819.1)
Qinghai	498.08 (403.61, 594.22)	1853.91 (1617.8, 2084.28)	613.94 (485.1, 775.7)
Ningxia	564.65 (456.21, 685.23)	1050.16 (914.07, 1191.46)	498.91 (380.33, 650.95)
Xinjiang	495.17 (409.58, 586.49)	1776.78 (1506.77, 2033.21)	890.89 (689.62, 1121.79)

Table S4 DALY rates (per 100 000) for different age and sexes groups in China in 2019.

Sex	Age group	Lung cancer	Chronic obstructive pulmonary disease	Diabetes mellitus
Male	<5 years	0	5 (3, 6)	0
Male	5-9 years	0	11 (7, 15)	0
Male	10-14 years	3 (2, 4)	16 (11, 22)	0
Male	15-19 years	19 (15, 25)	32 (24, 39)	12 (7, 21)
Male	20-24 years	41 (31, 53)	48 (37, 60)	62 (38, 97)
Male	25-29 years	71 (57, 88)	59 (47, 73)	133 (81, 202)
Male	30-34 years	159 (129, 194)	87 (71, 104)	230 (151, 336)
Male	35-39 years	311 (247, 387)	125 (101, 149)	359 (249, 505)
Male	40-44 years	605 (466, 767)	221 (179, 265)	526 (373, 721)
Male	45-49 years	1028 (771, 1343)	365 (293, 445)	734 (539, 976)
Male	50-54 years	1923 (1448, 2489)	714 (568, 876)	983 (748, 1277)
Male	55-59 years	3183 (2412, 4102)	1267 (1028, 1539)	1271 (974, 1613)
Male	60-64 years	4750 (3662, 6041)	2362 (1929, 2946)	1576 (1200, 1985)
Male	65-69 years	6188 (4843, 7706)	4230 (3477, 5188)	1985 (1540, 2487)
Male	70-74 years	7888 (6276, 9752)	8598 (7051, 10594)	2513 (2002, 3077)
Male	75-79 years	8251 (6599, 9974)	13899 (11482, 16600)	2830 (2301, 3409)
Male	80+ years	8017 (6691, 9377)	26234 (22519, 30385)	3285 (2761, 3826)
Female	<5 years	0	7 (4, 9)	0
Female	5-9 years	0	17 (12, 22)	0
Female	10-14 years	3 (2, 4)	27 (20, 35)	0
Female	15-19 years	13 (11, 16)	44 (33, 56)	10 (6, 16)
Female	20-24 years	26 (20, 33)	63 (47, 79)	43 (27, 68)
Female	25-29 years	44 (33, 57)	79 (59, 101)	84 (51, 129)
Female	30-34 years	93 (70, 120)	104 (80, 132)	133 (83, 200)
Female	35-39 years	183 (140, 231)	136 (106, 170)	204 (135, 295)
Female	40-44 years	358 (280, 449)	199 (158, 245)	312 (215, 436)
Female	45-49 years	509 (396, 641)	316 (255, 388)	502 (360, 674)
Female	50-54 years	844 (655, 1065)	559 (456, 694)	779 (581, 1023)
Female	55-59 years	1258 (988, 1579)	926 (754, 1156)	1121 (858, 1454)
Female	60-64 years	1850 (1474, 2318)	1645 (1343, 2141)	1547 (1213, 1961)
Female	65-69 years	2387 (1931, 2904)	2937 (2404, 3744)	2013 (1605, 2501)
Female	70-74 years	2994 (2462, 3595)	5645 (4645, 7314)	2479 (2018, 3030)
Female	75-79 years	3259 (2666, 3898)	9066 (7477, 11531)	2653 (2165, 3206)
Female	80+ years	2956 (2372, 3509)	16591 (13454, 21230)	2498 (2037, 3001)
Both	All ages	1204 (1008, 1422)	1401 (1220, 1667)	675 (534, 837)

Table S5 Population (unit: thousand) and per capita Gross Domestic Product per year (unit: CNY)[6] in urban areas of 330 Chinese city in 2019.

City	Province	Population	GDPp	City	Province	Population	GDPp	City	Province	Population	GDPp
Beijing	Beijing	18654	164220	Wuhu	Anhui	2477	96154	Guilin	Guangxi	1870	41294
Tianjin	Tianjin	13040	90371	Suzhou	Anhui	3166	34773	Hechi	Guangxi	1423	24703
Baoding	Hebei	5916	31856	Xuancheng	Anhui	1411	58819	Hezhou	Guangxi	1716	33676
Cangzhou	Hebei	1208	47663	Fuzhou	Fujian	6115	120879	Laibin	Guangxi	1605	29215
Chengde	Hebei	1228	41080	Longyan	Fujian	2256	101476	Liuzhou	Guangxi	2582	77056
Handan	Hebei	7820	36546	Nanping	Fujian	1813	74036	Nanning	Guangxi	5554	61738
Hengshui	Hebei	2088	33599	Ningde	Fujian	1096	84251	Qinzhou	Guangxi	2149	40922
Langfang	Hebei	1801	65512	Putian	Fujian	5187	89342	Wuzhou	Guangxi	1130	32303
Qinhuangdao	Hebei	3009	51334	Quanzhou	Fujian	2488	114067	Yulin	Guangxi	1605	28647
Shijiazhuang	Hebei	8741	52859	Sanming	Fujian	611	100641	Haikou	Hainan	4164	72218
Tangshan	Hebei	6858	86667	Xiamen	Fujian	5503	142739	Sanyan	Hainan	1433	87105
Xingtai	Hebei	1883	28707	Zhangzhou	Fujian	1349	92075	Chongqing	Chongqing	20868	75828
Zhangjiakou	Hebei	3193	35025	Fuzhou	Jiangxi	3040	37272	Chengdu	Sichuan	11501	103386
Datong	Shanxi	3245	38122	Ganzhou	Jiangxi	4082	39968	Dazhou	Sichuan	2311	35625
Jincheng	Shanxi	811	57714	Jian	Jiangxi	1060	42060	Deyang	Sichuan	1234	65745
Jinzhong	Shanxi	1298	43125	Jingdezhen	Jiangxi	848	55228	Ganmei	Sichuan	512	32440
Linfen	Shanxi	1663	32250	Jiujiang	Jiangxi	1803	63584	Guangan	Sichuan	1654	38522
Lvliang	Shanxi	588	38890	Nanchang	Jiangxi	5549	100415	Guangyuan	Sichuan	1208	35262
Suzhou	Shanxi	1379	59552	Pingxiang	Jiangxi	1555	48007	Leshan	Sichuan	1523	56999
Taiyuan	Shanxi	6084	90698	Shangrao	Jiangxi	4047	36839	Liangshan	Sichuan	2363	34085
Xinzhou	Shanxi	1115	31573	Xinyu	Jiangxi	1608	81641	Luzhou	Sichuan	1996	48105
Yangquan	Shanxi	1399	50775	Yichun	Jiangxi	2068	48182	Meishan	Sichuan	1575	46168
Yuncheng	Shanxi	1440	29126	Yingtan	Jiangxi	1131	79883	Mianyang	Sichuan	2297	58685
Changzhi	Shanxi	3184	47219	Binzhou	Shandong	1784	62639	Nanchong	Sichuan	2547	36073
Alxa League	Inner Mongolia	376	118101	Dezhou	Shandong	2025	52295	Neijiang	Sichuan	1825	38743
Bayan Nur	Inner Mongolia	978	51722	Dongying	Shandong	1816	134022	Panzhihua	Sichuan	853	82460
Baitou	Inner Mongolia	2954	93835	Heze	Shandong	3809	38867	Suining	Sichuan	1917	42113
Chifeng	Inner Mongolia	2408	39488	Jinan	Shandong	11170	106416	Yanan	Sichuan	814	46984
Ordos	Inner Mongolia	583	173069	Jining	Shandong	3070	52331	Yinbin	Sichuan	3020	57003
Hohhot	Inner Mongolia	2653	89138	Laiwu	Shandong	0	0	Ziyang	Sichuan	1405	31019
Hulun Buir	Inner Mongolia	696	47116	Liaocheng	Shandong	3005	37129	Zigong	Sichuan	1930	48904
Tongliao	Inner Mongolia	1580	40410	Linyi	Shandong	4597	43213	Bijie	Guizhou	2185	33156
Wuhai	Inner Mongolia	828	97565	Qingdao	Shandong	8502	124282	Guiyang	Guizhou	3452	81995
Ulanqab	Inner Mongolia	602	38622	Rizhao	Shandong	2250	66313	Liupanshui	Guizhou	646	43003
Xilingol League	Inner Mongolia	1336	75585	Taian	Shandong	2780	47248	Qiandongnan	Guizhou	2327	46048
Hinggan League	Inner Mongolia	1110	32310	Weihai	Shandong	2202	104615	Qiannan	Guizhou	2133	31678
Anshan	Liaoning	2046	49123	Weifang	Shandong	3150	60760	Tongren	Guizhou	659	39298
Benxi	Liaoning	1233	46673	Yantai	Shandong	3118	107343	Zunyi	Guizhou	2935	55411
Chaoyang	Liaoning	855	28761	Zaozhuang	Shandong	4002	43100	Baoshan	Yunnan	1425	36548
Dalian	Liaoning	5676	99996	Zibo	Shandong	4661	77510	Chuxiong	Yunnan	1638	45499
Dandong	Liaoning	1079	32256	Anyang	Henan	2506	43002	Dali	Yunnan	2168	38095

Fushun	Liaoning	1892	41382	Hebi	Henan	1369	60678	Dehong	Yunnan	970	38914
Fuxin	Liaoning	1037	27945	Jiaozuo	Henan	2064	76828	Diqing	Yunnan	182	61690
Huludao	Liaoning	1359	31802	Kaifeng	Henan	3622	51733	Honghe	Yunnan	3245	46475
Jinzhou	Liaoning	1331	35431	Luoyang	Henan	4401	72912	Kunming	Yunnan	4928	93853
Liaoyang	Liaoning	1191	45498	Luohe	Henan	2843	59190	Lijiang	Yunnan	243	36369
Panjin	Liaoning	1444	88983	Nanyang	Henan	4296	38064	Lincang	Yunnan	500	29926
Shenyang	Liaoning	8591	77777	Pingdingshan	Henan	2338	47201	Nujiang	Yunnan	440	34686
Tieling	Liaoning	589	24382	Puyang	Henan	1579	43810	Puer	Yunnan	364	33097
Yingkou	Liaoning	1317	54545	Sanxiamen	Henan	1327	63473	Qujing	Yunnan	2153	42774
Baicheng	Jilin	697	25980	Shangqiu	Henan	3980	39719	Wenshan	Yunnan	1971	29528
Baishan	Jilin	755	43744	Xinxiang	Henan	2317	50277	Xishuangbannan	Yunnan	925	47659
Jilin	Jilin	2614	34335	Xinyang	Henan	3327	42641	Yuxi	Yunnan	1137	81667
Liaoyuan	Jilin	653	35108	Xuchang	Henan	2843	76312	Zhaotong	Yunnan	1456	21255
Siping	Jilin	973	24979	Zhengzhou	Henan	8361	113139	Ali	Tibet	66	55591
Songyuan	Jilin	813	26559	Zhoukou	Henan	1348	36891	Changdu	Tibet	157	27844
Tonghua	Jilin	624	33702	Zhumadian	Henan	1811	38943	Lhasa	Tibet	407	86750
Yanbian	Jilin	2091	34789	Ezhou	Hubei	1889	106678	Linzhi	Tibet	66	73224
Changchun	Jilin	6462	78456	Enshi	Hubei	2614	34259	Naqu	Tibet	144	30420
Daqing	Heilongjiang	2206	94289	Huanggang	Hubei	590	36685	Rikaze	Tibet	171	69014
Da Hinggan Ling	Heilongjiang	580	30700	Huangshi	Hubei	1046	71511	Shannan	Tibet	92	49550
Haerbin	Heilongjiang	8906	48559	Jingmen	Hubei	1096	70203	Ankang	Shaanxi	1490	44241
Hegang	Heilongjiang	966	33981	Jingzhou	Hubei	1822	45097	Baiji	Shaanxi	2030	59050
Heihe	Heilongjiang	290	36478	Shiyan	Hubei	2007	59163	Hanzhong	Shaanxi	1665	45033
Jixi	Heilongjiang	1224	32278	Suizhou	Hubei	1113	52380	Shangluo	Shaanxi	818	35181
Jiamusi	Heilongjiang	1224	32788	Wuhan	Hubei	15282	145545	Tongchuan	Shaanxi	1022	44794
Mudanjiang	Heilongjiang	1385	32811	Xianning	Hubei	1063	62650	Weinan	Shaanxi	1388	34481
Qitaihe	Heilongjiang	757	29912	Xiangyang	Hubei	3846	84815	Xian	Shaanxi	11992	92256
Qiqihar	Heilongjiang	2110	22667	Xiaogan	Hubei	1619	46772	Xianyang	Shaanxi	803	50338
Shuangyashan	Heilongjiang	741	33857	Yichang	Hubei	2159	107830	Yanan	Shaanxi	979	73703
Suihua	Heilongjiang	1305	21045	Changde	Hunan	3450	62493	Yulin	Shaanxi	847	120908
Yichun	Heilongjiang	1143	26384	Chenzhou	Hunan	1957	50760	Baiyin	Gansu	679	27990
Shanghai	Shanghai	21439	157279	Hengyang	Hunan	2471	46379	Dingxi	Gansu	638	14746
Changzhou	Jiangsu	4715	156390	Huaihua	Hunan	979	32453	Gannan	Gansu	367	30252
Huaian	Jiangsu	5147	78543	Loudi	Hunan	1517	41675	Jiayuguan	Gansu	407	112219
Lianyungang	Jiangsu	3467	69523	Shaoyang	Hunan	1688	29339	Jinchang	Gansu	285	73437
Nanjing	Jiangsu	10940	165682	Xiangtan	Hunan	2104	78575	Jingquan	Gansu	557	54729
Nantong	Jiangsu	3313	128294	Xiangxi	Hunan	3083	26691	Lanzhou	Gansu	2879	75217
Suzhou	Jiangsu	5778	179174	Yiyang	Hunan	3303	40578	Linxia	Gansu	1046	14697
Taizhou	Jiangsu	2527	110731	Yongzhou	Hunan	2862	37013	Longnan	Gansu	815	16868
Wuxi	Jiangsu	4130	180044	Yueyang	Hunan	2716	65357	Pingliang	Gansu	720	21514
Suqian	Jiangsu	2743	62840	Zhangjiajie	Hunan	1321	35767	Qingyang	Gansu	543	32690
Xuzhou	Jiangsu	5285	81138	Changsha	Hunan	8905	139877	Tianshui	Gansu	1793	18819
Yancheng	Jiangsu	3760	79149	Zhuzhou	Hunan	3229	74618	Wuwei	Gansu	1413	26744
Yangzhou	Jiangsu	3590	128856	Chaozhou	Guangdong	2450	40664	Zhangye	Gansu	706	36314

Zhenjiang	Jiangsu	1587	128981	Dongguan	Guangdong	11305	112507	Guoluo	Qinghai	100	21983
Hangzhou	Zhejiang	13293	152465	Foshan	Guangdong	6682	133850	Haibei	Qinghai	150	32227
Huzhou	Zhejiang	2266	102593	Guangzhou	Guangdong	13827	156427	Hainan	Qinghai	226	36604
Jainxing	Zhejiang	1902	112751	Heyuan	Guangdong	464	34842	Hiaxi	Qinghai	451	128172
Jinhua	Zhejiang	2023	81224	Huizhou	Guangdong	2479	86043	Huangnan	Qinghai	138	36243
Lishui	Zhejiang	850	66936	Jiangmen	Guangdong	2116	68194	Xining	Qinghai	1267	55812
Ningbo	Zhejiang	6090	143157	Jieyang	Guangdong	3073	34471	Yushuo	Qinghai	276	14256
Quzhou	Zhejiang	1720	71087	Maoming	Guangdong	4421	51119	Guyuan	Ningxia	642	25886
Shaoxing	Zhejiang	4532	114561	Meizhou	Guangdong	1406	27096	Shizuishan	Ningxia	614	63494
Taizhou	Zhejiang	3298	83555	Qingyuan	Guangdong	2160	43770	Wuzhong	Ningxia	572	40889
Wenzhou	Zhejiang	3520	71225	Shantou	Guangdong	8175	47669	Yinchuan	Ningxia	1745	83492
Panshan	Zhejiang	1457	116781	Shanwei	Guangdong	754	35958	Zhongwei	Ningxia	586	37358
Anqing	Anhui	1214	50574	Shaoguan	Guangdong	1333	43743	Aksu	Xinjiang	1181	42531
Bangfu	Anhui	1903	60469	Shenzhen	Guangdong	7986	203489	Altay	Xinjiang	349	51524
Haozhou	Anhui	2788	33314	Yangjiang	Guangdong	1797	50412	Bozhou	Xinjiang	282	74276
Chizhou	Anhui	1099	56217	Yunfu	Guangdong	1000	36354	Changji	Xinjiang	832	82605
Chuzhou	Anhui	919	70429	Zhanjiang	Guangdong	2464	41720	Hami	Xinjiang	577	98148
Fuyang	Anhui	3805	32855	Fuqing	Guangdong	2116	53936	Hetian	Xinjiang	738	14923
Hefei	Anhui	4773	115623	Zhongshan	Guangdong	4334	92709	Kashi	Xinjiang	1436	22647
Huaibei	Anhui	1722	47654	Zhuhai	Guangdong	1928	175533	Karamay	Xinjiang	416	18857
Huainan	Anhui	3002	37140	Baise	Guangxi	516	34194	Kezhou	Xinjiang	215	25556
Huangshan	Anhui	771	57853	Beihai	Guangxi	977	76955	Tacheng	Xinjiang	523	54095
Liuan	Anhui	3641	33370	Chongzuo	Guangxi	530	36129	Turopan	Xinjiang	389	60985
Maanshan	Anhui	1345	89867	Fangchenggang	Guangxi	823	73163	Urumqi	Xinjiang	2979	96723
Tongling	Anhui	1493	58726	Guigang	Guangxi	2861	28451	Ili Kazak	Xinjiang	1718	42768

Table S6 Proportion for populations with different ages and genders in 31 Chinese provinces.

Age (years)	<5	5-9	10-14	15-19	20-24	25-29	30-34	35-39	40-44	45-49	50-54	55-59	60-64	65-69	70-74	75-79	80+	<5	5-9	10-14	15-19	20-24	25-29	30-34	35-39	40-44	45-49	50-54	55-59	60-64	65-69	70-74	75-79	80+
Sex (M: males; F: females)	M	M	M	M	M	M	M	M	M	M	M	M	M	M	M	M	M	F	F	F	F	F	F	F	F	F	F	F	F	F	F	F	F	F
Beijing	0.025	0.024	0.016	0.017	0.033	0.046	0.059	0.052	0.039	0.038	0.036	0.036	0.031	0.026	0.014	0.008	0.004	0.023	0.022	0.015	0.014	0.031	0.044	0.057	0.051	0.037	0.036	0.035	0.036	0.033	0.029	0.016	0.011	0.005
Tianjin	0.024	0.025	0.021	0.026	0.035	0.037	0.055	0.048	0.036	0.037	0.036	0.039	0.035	0.030	0.018	0.010	0.004	0.022	0.023	0.019	0.021	0.030	0.033	0.050	0.045	0.034	0.034	0.034	0.037	0.036	0.032	0.019	0.012	0.005
Hebei	0.031	0.035	0.030	0.036	0.027	0.031	0.052	0.044	0.036	0.036	0.036	0.031	0.026	0.023	0.014	0.008	0.003	0.028	0.032	0.027	0.033	0.027	0.031	0.054	0.045	0.036	0.037	0.037	0.033	0.027	0.025	0.016	0.010	0.004
Shanxi	0.029	0.031	0.029	0.031	0.031	0.038	0.050	0.043	0.036	0.042	0.039	0.034	0.026	0.020	0.011	0.007	0.003	0.027	0.029	0.027	0.031	0.034	0.039	0.051	0.043	0.036	0.043	0.039	0.033	0.025	0.020	0.012	0.009	0.003
Inner Mongolia	0.025	0.027	0.024	0.027	0.030	0.036	0.050	0.044	0.038	0.046	0.044	0.038	0.028	0.020	0.012	0.008	0.003	0.024	0.025	0.022	0.025	0.030	0.036	0.049	0.044	0.038	0.047	0.044	0.038	0.028	0.023	0.015	0.011	0.003
Liaoning	0.020	0.022	0.019	0.022	0.026	0.030	0.047	0.041	0.039	0.041	0.042	0.045	0.038	0.031	0.017	0.010	0.004	0.019	0.020	0.017	0.020	0.024	0.029	0.048	0.041	0.039	0.042	0.043	0.046	0.041	0.035	0.020	0.013	0.006
Jilin	0.019	0.022	0.022	0.024	0.023	0.030	0.047	0.040	0.041	0.044	0.046	0.043	0.034	0.028	0.016	0.009	0.003	0.018	0.020	0.020	0.023	0.023	0.031	0.048	0.040	0.042	0.046	0.048	0.045	0.037	0.033	0.020	0.013	0.004
Heilongjiang	0.015	0.019	0.020	0.025	0.028	0.028	0.043	0.039	0.040	0.049	0.048	0.046	0.035	0.028	0.016	0.009	0.004	0.014	0.017	0.019	0.024	0.027	0.028	0.043	0.039	0.040	0.049	0.049	0.047	0.038	0.032	0.020	0.014	0.005
Shanghai	0.019	0.021	0.015	0.016	0.033	0.048	0.058	0.049	0.039	0.036	0.035	0.035	0.037	0.034	0.021	0.010	0.005	0.017	0.019	0.014	0.013	0.029	0.044	0.054	0.047	0.037	0.034	0.033	0.034	0.038	0.035	0.021	0.011	0.008
Jiangsu	0.025	0.030	0.025	0.027	0.032	0.041	0.056	0.041	0.037	0.039	0.043	0.035	0.025	0.024	0.016	0.009	0.003	0.023	0.026	0.021	0.023	0.028	0.037	0.055	0.041	0.037	0.039	0.044	0.036	0.025	0.025	0.017	0.011	0.005
Zhejiang	0.026	0.027	0.022	0.028	0.038	0.047	0.058	0.047	0.042	0.045	0.042	0.034	0.023	0.019	0.013	0.007	0.003	0.023	0.023	0.019	0.024	0.033	0.041	0.053	0.043	0.039	0.042	0.040	0.033	0.023	0.020	0.013	0.007	0.004
Anhui	0.031	0.033	0.028	0.032	0.032	0.039	0.047	0.035	0.036	0.042	0.044	0.035	0.019	0.020	0.013	0.009	0.003	0.028	0.028	0.024	0.029	0.031	0.039	0.051	0.038	0.037	0.042	0.046	0.037	0.019	0.022	0.014	0.010	0.004
Fujian	0.033	0.037	0.029	0.031	0.034	0.043	0.057	0.047	0.040	0.043	0.036	0.029	0.020	0.016	0.010	0.006	0.002	0.028	0.031	0.025	0.027	0.031	0.039	0.056	0.046	0.038	0.041	0.036	0.029	0.021	0.018	0.011	0.006	0.003
Jiangxi	0.031	0.038	0.038	0.048	0.039	0.033	0.042	0.034	0.038	0.041	0.037	0.030	0.022	0.018	0.012	0.007	0.002	0.026	0.032	0.032	0.042	0.036	0.032	0.044	0.035	0.039	0.040	0.037	0.030	0.022	0.019	0.012	0.008	0.003
Shandong	0.037	0.034	0.029	0.030	0.029	0.028	0.054	0.042	0.037	0.041	0.040	0.033	0.025	0.022	0.013	0.008	0.003	0.033	0.029	0.025	0.028	0.029	0.027	0.054	0.042	0.037	0.040	0.040	0.034	0.026	0.024	0.015	0.010	0.004
Henan	0.032	0.038	0.036	0.043	0.032	0.031	0.050	0.035	0.034	0.039	0.038	0.030	0.021	0.019	0.012	0.007	0.002	0.029	0.033	0.030	0.038	0.033	0.032	0.054	0.037	0.036	0.039	0.039	0.031	0.022	0.021	0.014	0.009	0.004
Hubei	0.027	0.029	0.025	0.033	0.033	0.039	0.051	0.039	0.037	0.041	0.043	0.038	0.025	0.023	0.015	0.008	0.003	0.024	0.025	0.021	0.029	0.030	0.037	0.051	0.039	0.037	0.040	0.042	0.038	0.026	0.024	0.016	0.010	0.004
Hunan	0.030	0.034	0.030	0.039	0.033	0.029	0.048	0.039	0.034	0.041	0.042	0.035	0.022	0.021	0.014	0.008	0.003	0.026	0.030	0.026	0.036	0.033	0.030	0.052	0.041	0.035	0.041	0.043	0.036	0.022	0.022	0.015	0.009	0.003
Guangdong	0.031	0.032	0.025	0.029	0.047	0.059	0.067	0.054	0.043	0.044	0.036	0.026	0.016	0.013	0.008	0.004	0.002	0.027	0.027	0.021	0.023	0.038	0.048	0.055	0.046	0.037	0.039	0.033	0.024	0.016	0.014	0.009	0.005	0.002
Guangxi	0.036	0.040	0.032	0.044	0.033	0.034	0.048	0.045	0.040	0.039	0.034	0.027	0.019	0.017	0.011	0.006	0.002	0.032	0.034	0.027	0.042	0.035	0.033	0.049	0.045	0.039	0.037	0.033	0.029	0.020	0.018	0.011	0.007	0.003
Hainan	0.035	0.038	0.032	0.033	0.036	0.049	0.055	0.047	0.038	0.041	0.038	0.030	0.018	0.015	0.010	0.006	0.002	0.029	0.031	0.025	0.028	0.031	0.045	0.052	0.045	0.036	0.037	0.034	0.028	0.018	0.016	0.010	0.006	0.003
Chongqing	0.027	0.027	0.025	0.033	0.037	0.039	0.047	0.035	0.027	0.048	0.043	0.034	0.022	0.024	0.014	0.009	0.003	0.025	0.025	0.023	0.031	0.038	0.040	0.048	0.035	0.028	0.049	0.045	0.036	0.023	0.026	0.016	0.010	0.004
Sichuan	0.026	0.026	0.022	0.029	0.037	0.040	0.050	0.035	0.032	0.048	0.044	0.033	0.021	0.023	0.015	0.010	0.003	0.024	0.024	0.021	0.028	0.038	0.042	0.050	0.035	0.033	0.049	0.046	0.036	0.022	0.026	0.016	0.011	0.004
Guizhou	0.040	0.037	0.031	0.038	0.041	0.041	0.048	0.037	0.036	0.042	0.035	0.027	0.017	0.014	0.010	0.007	0.002	0.035	0.032	0.027	0.035	0.046	0.042	0.048	0.036	0.035	0.040	0.036	0.028	0.018	0.017	0.012	0.008	0.003
Yunnan	0.032	0.028	0.023	0.038	0.043	0.043	0.051	0.040	0.040	0.044	0.037	0.030	0.018	0.017	0.011	0.007	0.002	0.029	0.026	0.021	0.038	0.046	0.041	0.048	0.038	0.038	0.041	0.036	0.031	0.019	0.019	0.012	0.008	0.003
Tibet	0.024	0.027	0.023	0.044	0.044	0.055	0.064	0.052	0.045	0.052	0.044	0.028	0.015	0.011	0.007	0.004	0.001	0.022	0.025	0.022	0.045	0.036	0.046	0.053	0.044	0.038	0.040	0.033	0.022	0.014	0.010	0.007	0.005	0.001
Shaanxi	0.031	0.032	0.025	0.029	0.036	0.045	0.059	0.045	0.035	0.039	0.035	0.031	0.024	0.019	0.012	0.008	0.003	0.028	0.029	0.022	0.025	0.035	0.044	0.059	0.044	0.034	0.037	0.035	0.030	0.024	0.020	0.013	0.009	0.003
Gansu	0.032	0.030	0.027	0.030	0.033	0.040	0.050	0.039	0.034	0.045	0.044	0.034	0.021	0.020	0.012	0.008	0.003	0.029	0.027	0.024	0.027	0.034	0.041	0.051	0.039	0.034	0.043	0.043	0.035	0.022	0.020	0.014	0.011	0.003
Qinghai	0.028	0.028	0.026	0.028	0.035	0.044	0.050	0.037	0.040	0.049	0.050	0.038	0.021	0.016	0.010	0.007	0.002	0.027	0.026	0.024	0.026	0.033	0.041	0.047	0.035	0.038	0.046	0.048	0.036	0.020	0.017	0.012	0.011	0.003
Ningxia	0.034	0.033	0.031	0.033	0.033	0.040	0.049	0.042	0.040	0.043	0.038	0.029	0.019	0.016	0.010	0.007	0.002	0.031	0.030	0.028	0.032	0.036	0.042	0.051	0.041	0.039	0.042	0.039	0.029	0.019	0.018	0.012	0.009	0.002
Xinjiang	0.026	0.033	0.027	0.029	0.034	0.043	0.052	0.039	0.038	0.052	0.049	0.035	0.020	0.014	0.009	0.007	0.002	0.024	0.031	0.026	0.027	0.031	0.039	0.048	0.037	0.036	0.049	0.046	0.033	0.020	0.015	0.012	0.011	0.003

Table S7 Exposure factor (f_{exp}) of NO_2 in 31 provinces in China. [16]

	Mean	SD	P5	P25	P50	P75	P95
Beijing	0.50	0.15	0.28	0.39	0.48	0.60	0.79
Tianjin	0.47	0.15	0.26	0.37	0.45	0.56	0.77
Hebei	0.46	0.14	0.26	0.36	0.45	0.55	0.72
Shanxi	0.45	0.13	0.25	0.36	0.44	0.53	0.69
Inner Mongolia	0.45	0.13	0.25	0.35	0.43	0.53	0.69
Liaoning	0.42	0.15	0.20	0.31	0.40	0.51	0.69
Jilin	0.40	0.14	0.19	0.30	0.39	0.49	0.66
Heilongjiang	0.41	0.15	0.18	0.30	0.40	0.51	0.69
Shanghai	0.49	0.14	0.29	0.39	0.48	0.58	0.76
Jiangsu	0.47	0.13	0.27	0.37	0.45	0.55	0.71
Zhejiang	0.50	0.15	0.29	0.39	0.48	0.59	0.78
Anhui	0.48	0.14	0.28	0.38	0.47	0.57	0.75
Fujian	0.58	0.17	0.32	0.45	0.57	0.70	0.86
Jiangxi	0.57	0.16	0.32	0.44	0.56	0.69	0.87
Shandong	0.47	0.16	0.25	0.35	0.45	0.57	0.79
Henan	0.51	0.16	0.27	0.39	0.49	0.61	0.81
Hubei	0.52	0.14	0.32	0.42	0.51	0.61	0.77
Hunan	0.54	0.15	0.32	0.43	0.53	0.64	0.82
Guangdong	0.60	0.15	0.37	0.49	0.59	0.71	0.86
Guangxi	0.54	0.14	0.33	0.44	0.53	0.63	0.81
Hainan	0.62	0.15	0.38	0.51	0.62	0.74	0.88
Chongqing	0.56	0.14	0.36	0.46	0.55	0.65	0.82
Sichuan	0.54	0.14	0.33	0.44	0.52	0.62	0.80
Guizhou	0.51	0.15	0.29	0.40	0.49	0.60	0.80
Yunnan	0.53	0.13	0.33	0.43	0.52	0.61	0.76
Tibet	0.47	0.13	0.28	0.38	0.46	0.55	0.70
Shaanxi	0.48	0.13	0.29	0.39	0.47	0.56	0.73
Gansu	0.49	0.14	0.28	0.39	0.47	0.57	0.74
Qinghai	0.47	0.13	0.27	0.37	0.45	0.54	0.71
Ningxia	0.50	0.16	0.27	0.39	0.48	0.59	0.80
Xinjiang	0.48	0.13	0.27	0.38	0.47	0.55	0.71

References

[1] Y. Hu, B. Zhao, Indoor sources strongly contribute to exposure of Chinese urban residents to PM2.5 and NO2, J. Hazard. Mater. 426 (2022) 127829.

[2] C. Dimitroulopoulou, M.R. Ashmore, M.T.R. Hill, M.A. Byrne, R. Kinnersley, INDAIR: A probabilistic model of indoor air pollution in UK homes, Atmos. Environ. 40(33) (2006) 6362-6379.

[3] National Bureau of Statistics of China, China Statistical Yearbook-2020, China Statistics Press2020.

[4] M. Zhou, H. Wang, X. Zeng, P. Yin, J. Zhu, W. Chen, X. Li, L. Wang, L. Wang, Y. Liu, J. Liu, M. Zhang, J. Qi, S. Yu, A. Afshin, E. Gakidou, S. Glenn, V.S. Krish, M.K. Miller-Petrie, W.C. Mountjoy-Venning, E.C. Mullany, S.B. Redford, H. Liu, M. Naghavi, S.I. Hay, L. Wang, C.J.L. Murray, X. Liang, Mortality, morbidity, and risk factors in China and its provinces, 1990–2017: a systematic analysis for the Global Burden of Disease Study 2017, The Lancet 394(10204) (2019) 1145-1158.

[5] Global Burden of Disease Collaborative Network, Global Burden of Disease Study 2019 (GBD 2019) Results., Seattle, United States: Institute for Health Metrics and Evaluation (IHME),, 2020.

[6] Editorial board and editorial staff editorial board, China city statistical year book-2020, China Statistics Press2020.

[7] National Bureau of Statistics of China, Tabulation on the 2019 population census of the People's Republic of China (in Chinese), 2022. http://www.gov.cn/guoqing/2021-05/13/content_5606149.htm. (Accessed March 16 2022).

[8] E. Koehler, E. Brown, S.J.P.A. Haneuse, On the Assessment of Monte Carlo Error in Simulation-Based Statistical Analyses, American Statistician 63(2) (2009) 155-162.

[9] B. Zhou, B. Zhao, Population inhalation exposure to polycyclic aromatic hydrocarbons and associated lung cancer risk in Beijing region: Contributions of indoor and outdoor sources and exposures, Atmos. Environ. 62 (2012) 472-480.

[10] D. Spiegelhalter, A. Thomas, N. Best, D. Lunn, Tutorial: How many iterations after convergence?, Institute of Public Health, Cambridge, UK, 2003.

[11] P. Guo, K. Yokoyama, F. Piao, K. Sakai, M. Khalequzzaman, M. Kamijima, T. Nakajima, F. Kitamura, Sick Building Syndrome by Indoor Air Pollution in Dalian, China, Int. J. Environ. Res. Public. Health 10(4) (2013) 1489-1504.

[12] Y. Jiang, Y. Niu, Y. Xia, C. Liu, Z. Lin, W. Wang, Y. Ge, X. Lei, C. Wang, J. Cai, R. Chen, H. Kan, Effects of personal nitrogen dioxide exposure on airway inflammation and lung function, Environ. Res. 177 (2019).

[13] D. Li, Y. Wang, C. Xu, X. Zhang, R. Lu, B. Wang, X. Yao, X. Wang, J. Wang, Residential Indoor Air Pollution in Qingdao, J. Environ. Hyg. 10(03) (2020) 243-248+266.

[14] Q. Zhang, L. Hong, J. Peng, Z. Zhang, X. Zhang, D. Xie, Investigation on Indoor Air Pollution of Ｒesidents Ｒesidentiol District in Shenzhen at Xixiang, J. Environ. Hyg. 9(06) (2019) 545-549+556.

[15] H. Yin, C. Liu, L. Zhang, A. Li, Z. Ma, Measurement and evaluation of indoor air quality in naturally ventilated residential buildings, Indoor Built Environ. 28(10) (2019) 1307-1323.

[16] Y. Hu, M. Yao, Y. Liu, B. Zhao, Personal exposure to ambient PM2.5, PM10, O3, NO2, and SO2 for different populations in 31 Chinese provinces, Environ. Int. 144 (2020) 106018.
